# Supplementary material for: Differential human antibody repertoires following Zika infection and the implications for serodiagnostics and disease outcome
Source: Nat Commun. 2019 Apr 26;10:1943. doi: 10.1038/s41467-019-09914-3 (PMC6486612; doi:10.1038/s41467-019-09914-3)
Supplement: Supplementary file 1 — Supplementary Information [file 41467_2019_9914_MOESM1_ESM.pdf]

Supplementary Information

Differential human antibody repertoires following Zika infection and the implications for Serodiagnostics and disease outcome

Ravichandran et al

```
10 20 30 40 50 60 70 80 90 100 110 120
MKNPKKKSGGFRIVNMLKRGVARVSPFGGLKRLPAGLLLGHGPIRMVLAILAFLRFTAIKPSLGLINRWGSVGKKEAMEIIKKFKKDLAAMLRIINARKEKKRRGADTSVGIVGLLLTTA
________C________>

130 140 150 160 170 180 190 200 210 220 230 240
MAAEVTRRGSAYYMYLDRNDAGEAISFPTTLGMNKCYIQIMDLGHMCDATMSYECPMLDEGVEPDDVDCWCNTTSTWVVYGTCHHKKGEARRSRRAVTLPSHSTRKLQTRSQTWLESREY
________PR________>
________M________>

250 260 270 280 290 300 310 320 330 340 350 360
TKHLIRVENWIFRNPGFALAAAAIAWLLGSSTSQKVIYLVMILLIAPAYSIRCIGVSNRDFVEGMSGGTWVDVVLEHGGCVTVMAQDKPTVDIELVTTTVSNMAEVRSYCYEASISDMAS
________M________>
________E________>

370 380 390 400 410 420 430 440 450 460 470 480
DSRCPTQGEAYLDKQSDTQYVCKRTLVDRGWGNGCGLFGKGSLVTCAKFACSKKMTGKSIQPENLEYRIMLSVHGSQHSGMIVNDTGHETDENRAKVEITPNSPRAEATLGGFGSLGLDC
________E________>

490 500 510 520 530 540 550 560 570 580 590 600
EPRTGLDFSDLYYLTMNNKHWLVHKEWFHDIPLPWHAGADTGTPHWNNKEALVEFKDAHAKRQTVVVLGSQEGAVHTALAGALEAEMDGAKGRLSSGHLKCRLKMDKLRLKGVSYSLCTA
________E________>

610 620 630 640 650 660 670 680 690 700 710 720
AFTFTKIPAETLHGTVTVEVQYAGTDGPCKVPAQMAVDMQTLTPVGRLITANPVITESTENSKMMLELDPPFGDSYIVIGVGEKKITHHWHRSGSTIGKAFEATVRGAKRMAVLGDTAWD
________E________>

730 740 750 760 770 780 790 800 810 820 830 840
FGSVGGALNSLGKGIHQIFGAAFKSLFGGMSWFSQILIGTLLMWLGLNTKNGSISLMCLALGGVLIFLSTAVSADVGCSVDFSKKETRCGTGVFVYNDVEAWRDRYKYHPDSPRRLAAAV
________E________>
________NS1________>

850 860 870 880 890 900 910 920 930 940 950 960
KQAWEDGICGISSVSRMENIMWRSVEGELNAILEENGVQLTVVVGSVKNPMWRGPQRLPVPVNELPHGWKAWGKSYFVRAAKTNNSFVVDGDTLKECPLKHRAWNSFLVEDHGFGVFHTS
________NS1________>

970 980 990 1000 1010 1020 1030 1040 1050 1060 1070 1080
VWLKVREDYSLECDPAVIGTAVKGKEAVHSDLGYWIESEKNDTWRLKRAHLIEMKTCEWPKSHTLWTDGIEESDLIIPKSLAGPLSHHNTREGYRTQMKGPWHSEELEIRFEECPGTKVH
________NS1________>

1090 1100 1110 1120 1130 1140 1150 1160 1170 1180 1190 1200
VEETCGTRGPSLRSTTASGRVIEEWCCRECTMPPLSFRAKDGCWYGMEIRPRKEPESNLVRSVVTAGSTDHMDHFSLGVLVILLMVQEGLKKRMTTKIIISTSMAVLVAMILGGFSMSDL
________NS1________>
________NS2A________>

1210 1220 1230 1240 1250 1260 1270 1280 1290 1300 1310 1320
AKLAILMGATFAEMNTGGDVAHLALIAAFKVRPALLVSFIFRANWTPRESMLLALASCLLQTAISALEGDLMVLINGFALAWLAIRAMVVPRTDNITLAILAALTPLARGTLLVAWRAGL
________NS2A________>

1330 1340 1350 1360 1370 1380 1390 1400 1410 1420 1430 1440
ATCGGFMLLSLKGKGSVKKNLPFVMALGLTAVRLVDPINVVGLLLLTRSGKRSWPPSEVLTAVGLICALAGGFAKADIEMAGPMAAVGLLIVSYVVSGKSVDMYIERAGDITWEKDAEVT
________NS2A________>
________NS2B________>

1450 1460 1470 1480 1490 1500 1510 1520 1530 1540 1550 1560
GNSPRLDVALDESGDFSLVEDDGPPMREIILKVVLMTICGMNPIAIPFAAGAWYVYVKTGKRSGALWDVPAPKEVKKGETTDGVYRVMTRRLLGSTQVGVGVMQEGVFHTMWHVTKGSAL
________NS2B________>
________NS3________>

1570 1580 1590 1600 1610 1620 1630 1640 1650 1660 1670 1680
RSGEGRLDPYWGDVKQDLVSYCGPWKLDAAWDGHSEVQLLAVPPGERARNIQTLPGIFKTKDGDIGAVALDYPAGTSGSPILDKCGRVIGLYGNGVVIKNGSYVSAITQGRREEETPVEC
________NS3________>

1690 1700 1710 1720 1730 1740 1750 1760 1770 1780 1790 1800
FEPSMLKKKQLTVLDLHPGAGKTRRVLPEIVREAIKTRLRTVILAPTRVVAAEMEEALRGLPVRYMTTAVNVTHSGTEIVDLMCHATFTSRLLQPIRVPNYNLYIMDEAHFTDPSSIAAR
________NS3________>

1810 1820 1830 1840 1850 1860 1870 1880 1890 1900 1910 1920
GYISTRVEMGEAAAIFMTATPPGTRDAFPDSNSPIMDTEVEVPERAWSSGFDWVTDHSGKTVWFVPSVRNGNEIAACLTKAGKRVIQLSRKTFETEFQKTKHQEWDFVVTTDISEMGANF
________NS3________>

1930 1940 1950 1960 1970 1980 1990 2000 2010 2020 2030 2040
KADRVIDSRRCLKPVILDGERVILAGPMPVTHASAAQRRGRIGRNPNKPGDEYLYGGGCAETDEDHAHWLEARMLLDNIYLQDGLIASLYRPEADKVAAIEGEFKLRTEQRKTFVELMKR
________NS3________>

2050 2060 2070 2080 2090 2100 2110 2120 2130 2140 2150 2160
GDLPVWLAYQVASAGITYTDRRWCFDGTTNNTIMEDSVPAEVWTRHGEKRVLKPRWMDARVCSDHAALKSFKEFAAGKRGAAFGVMEALGTLPGHMTERFQEAIDNLAVLMRAETGSRPY
________NS3________>
________NS4A________>

2170 2180 2190 2200 2210 2220 2230 2240 2250 2260 2270 2280
KAAAAQLPETLETIMLLGLLGTVSLGIFFVLMRNKGIGKMGFGMVTLGASAWLMWLSEIEPARIACVLIVVFLLLVVLIPEPEKQRSPQDNQMAIIIMVAVGLLGLITANELGWLERTKS
________NS4A________>

2290 2300 2310 2320 2330 2340 2350 2360 2370 2380 2390 2400
DLSHLMGRREEGATIGFSMDIDLRPASAWAIYAALTTFITPAVQHAVTTSYNNYSLMAMATQAGVLFGMGKGMPFYAWDFGVPLLMIGCYSQLTPLTLIVAIILLVAHYMYLIPGLQAAA
________NS4B________>

2410 2420 2430 2440 2450 2460 2470 2480 2490 2500 2510 2520
ARAAQKRTAAGIMKNPVVDGIVVTDIDTMTIDPQVEKKMGQVLLIAVAVSSAILSRTAWGWGEAGALITAATSTLWEGSPNKYWNSSTATSLCNIFRGSYLAGASLIYTVTRNAGLVKRR
________NS4B________>

2530 2540 2550 2560 2570 2580 2590 2600 2610 2620 2630 2640
GGGTGETLGEKWKARLNQMSALEFYSYKKSGITEVCREEARRALKDGVATGGHAVSRGSAKLRWLVERGYLQPYGKVIDLGCGRGGWSYYAATIRKVQEVKGYTKGGPGHEEPVLVQSYG
________NS5________>

2650 2660 2670 2680 2690 2700 2710 2720 2730 2740 2750 2760
WNIVRLKSGVDVFHMAAEPCDTLLCDIGESSSSPEVEEARTLRVLSMVGDWLEKRPGAFCIKVLCPYTSTMMETLERLQRRYGGGLVRVPLSRNSTHEMYWVSGAKSNTIKSVSTTSQLL
________NS5________>

2770 2780 2790 2800 2810 2820 2830 2840 2850 2860 2870 2880
LGRMDGPRRPVKYEEDVNLGSGTRAVVSCAEAPNMKIIGNRIERIRSEHAETWFFDENHPYRTWAYHGSYEAPTQGSASSLINGVVRLLSKPWDVVTGVTGIAMTDTTPYGQQRVFKEKV
________NS5________>

2890 2900 2910 2920 2930 2940 2950 2960 2970 2980 2990 3000
DTRVPDPQEGTRQVMSMVSSWLWKELGKHKRPRVCTKEEFINKVRSNAALGAIFEEEKEWKTAVEAVNDPRFWALVDKEREHHLRGECQSCVYNMMGKREKKQGEFGKAKGSRAIWYMWL
________NS5________>

3010 3020 3030 3040 3050 3060 3070 3080 3090 3100 3110 3120
GARFLEFEALGFLNEDHWMGRENSGGGVEGLGLQRLGYVLEEMSRIPGGRMYADDTAGWDTRISRFDLENEALITNQMEKGHRALALAIIKYTYQNKVVKVLRPAEKGKTVMDIISRQDQ
________NS5________>

3130 3140 3150 3160 3170 3180 3190 3200 3210 3220 3230 3240
RGSGQVVTYALNTFTNLVVQLIRNMEAEEVLEMQDLWLLRRSEKVTNWLQSNGWDRLKRMAVSGDDCVVKPIDDRFAHALRFLNDMGKVRKDTQEWKPSTGWDNWEEVPFCSHHFNKLHL
________NS5________>

3250 3260 3270 3280 3290 3300 3310 3320 3330 3340 3350 3360
KDGRSIVVPCRHQDELIGRARVSPGAGWSIRETACLAKSYAQMWQLLYFHRRDLRLMANAICSSVPVDWVPTGRTTWSIHGKGEWMTTEDMLVVWNRVWIEENDHMEDKTPVTKWTDIPY
________NS5________>

3370 3380 3390 3400 3410 3420
LGKREDLWCGSLIGHRPRTTWAENIKNTVNMVRRIIGDEEKYMDYLSTQVRYLGEEGSTPGVL
________NS5________>
```

Supplementary Figure 1: Complete ZIKV ICD Paraiba strain whole genome translated sequence used for construction of ZIKV GFPD library and depiction in Figures 1-3.

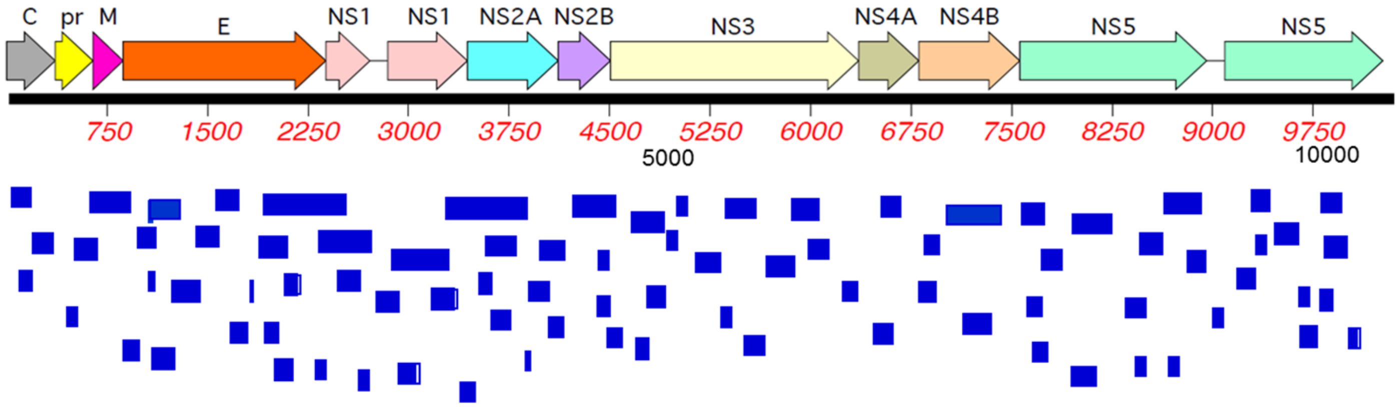

Supplementary Figure 2: Random distribution of size and sequence of the ZIKV-GFPDL. Sequencing of ZIKV whole genome fragments expressed by the phages of the ZIKV GFPD libraries were aligned to the ZIKV_ICD translated sequence (shown in Supplementary figure 1).

a

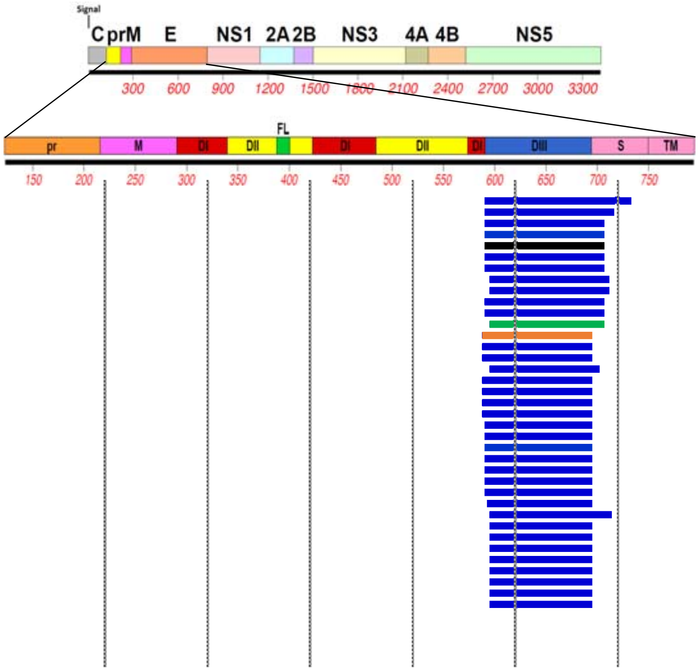

b

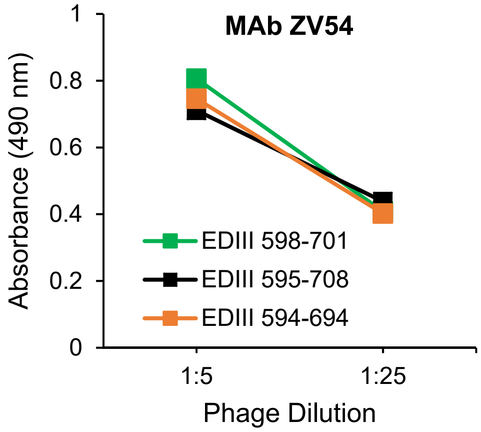

c

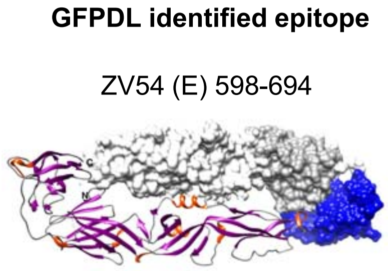

d

ZIKV MAb	Neutralizing Strains	Epitope	AA Sequence
ZV54	ZIKV Asian, African, and American strains (Zika H/PF/2013, MR766, Uganda 1947, Paraiba, Brazil 2015)	Known Site using X-ray crystallography (conformational epitope- lateral ridge of EDIII)	**589-** RLKGVSYSLCTAAFTFTKIPAETLHGTVTVE VQYAGTDGPCKVPAQMAVDMQTLTPVGRL ITANPVITESTENSKMMLELDPPFGDSYIVIG VGEKKITHHWHRSGSTI**-697**
		GFPDL	**598-** CTAAFTFTKIPAETLHGTVTVEVQYAGTDG PCKVPAQMAVDMQTLTPVGRLITANPVITE STENSKMMLELDPPFGDSYIVIGVGEKKITH HWHRSG**-694**

Supplementary Figure 3: GFPDL based epitope mapping of neutralizing MAb ZV54. a) GFPDL-based epitope mapping of neutralizing ZIKV mouse MAb ZV54 to prME. b) The ELISA reactivity of selected GFPDL identified phage clones to MAb ZV54 (highlighted in green, black and orange in a) was confirmed by phage ELISA. c) Structure of GFPDL-identified epitope on PDB#5JHM for mature E (595-694 residues of identified epitope 598-694 sequence are colored blue in the available structure). d) The minimal overlapping sequence for MAb ZV54 identified using GFPDL mapping (598-694) is shown in the table compared to the sequence previously identified 'known site' (Zhao et al., Cell, 2016).

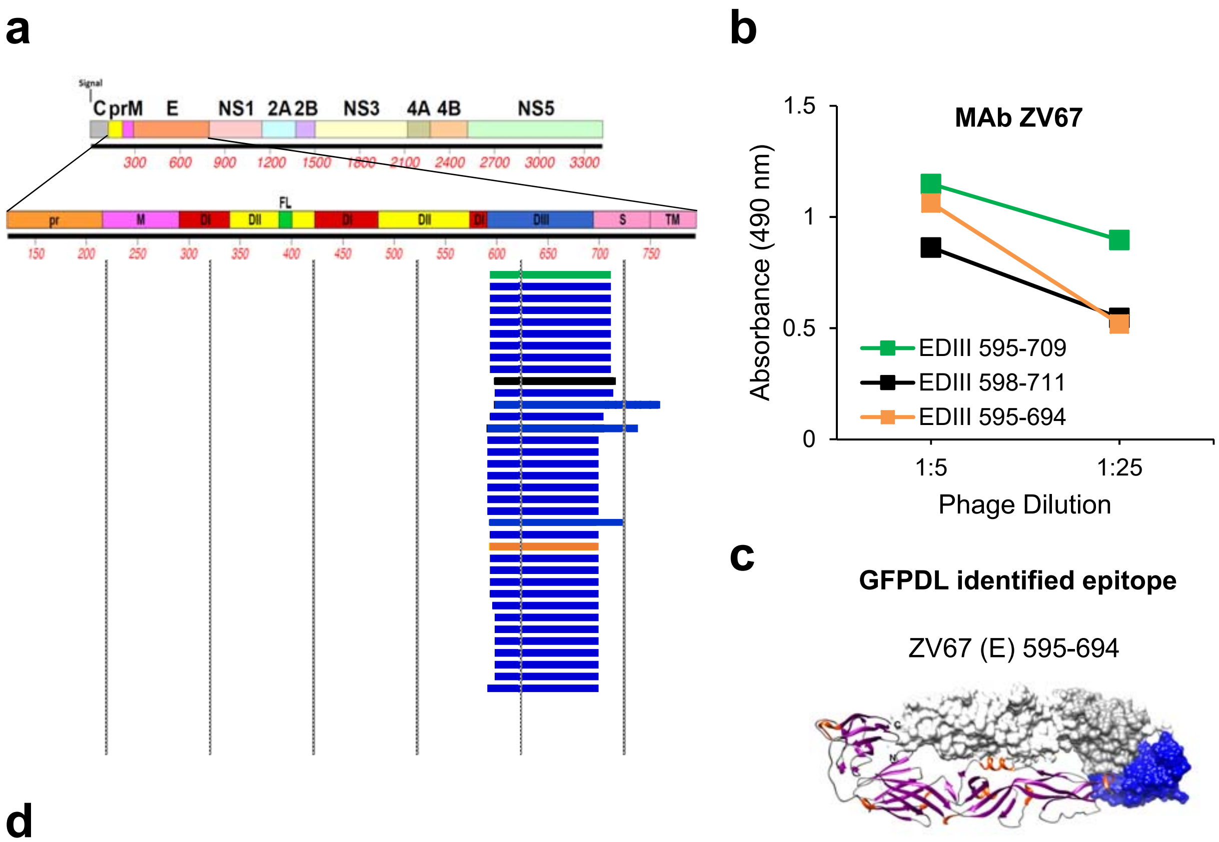

d

ZIKV MAb	Neutralizing Strains	Epitope	AA Sequence
ZV67	ZIKV Asian, African, and American strains (Zika H/PF/2013, MR766, Uganda 1947, Paraiba, Brazil 2015)	Known Site using X-ray crystallography (conformational epitope- lateral ridge of EDIII)	**589-** RLKGVSYSLCTAAFTFTKIPAETLHGTVTVE VQYAGTDGPCKVPAQMAVDMQTLTPVGR LITANPVITESTENSKMMLELDPPFGDSYIVI GVGEKKITHHWHRSGST**I-697**
		GFPDL	**595-** YSLCTAAFTFTKIPAETLHGTVTVEVQYAG TDGPCKVPAQMAVDMQTLTPVGRLITANP VITESTENSKMMLELDPPFGDSYIVIGVGE KKITHHWHRSG**-694**

Supplementary Figure 4: GFPDL based epitope mapping of neutralizing MAb ZV67. a) GFPDL-based epitope mapping of neutralizing ZIKV mouse MAb ZV67 to prME. b) The ELISA reactivity of selected GFPDL identified phage clones to MAb ZV67 (highlighted in green, black and orange in a) was confirmed by phage ELISA. c) Structure of GFPDL-identified epitope on PDB#5JHM for mature E (595-694 residues of identified epitope 595-694 sequence are colored blue in the available structure). d) The overlapping sequence for MAb ZV67 identified using GFPDL mapping (595-694) is shown in the table compared to the sequence previously identified 'known site' (Zhao et al., Cell, 2016).

a

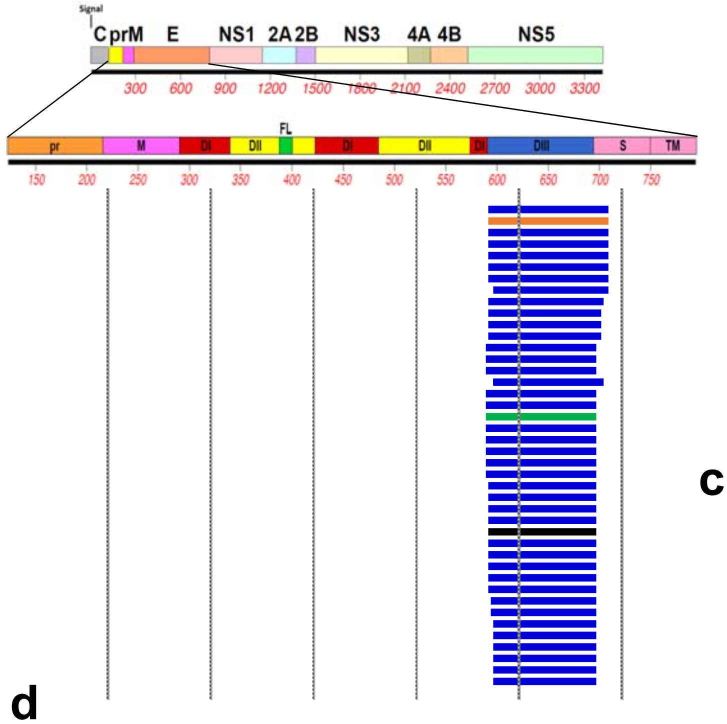

b

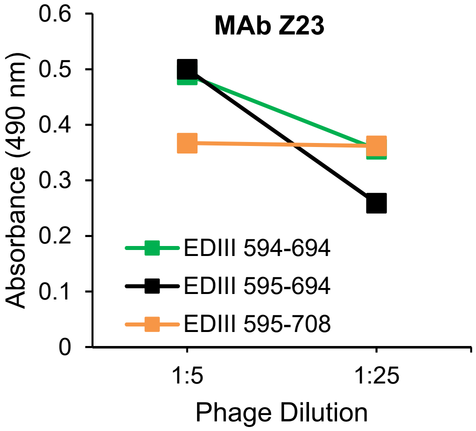

c

GFPDL identified epitope

Z23 (E) 594-694

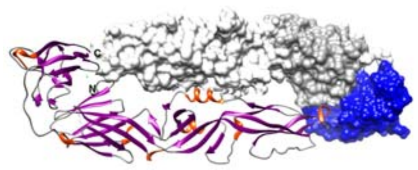

d

**ZIKV MAb**	**Neutralizing Strains**	**Epitope**	**AA Sequence**
Z23	ZIKV Asian strain SMGC-1	Known Site using crystallography	ZIKV E DIII tertiary epitope (Z23 mainly binds to DIII of one envelope protein monomer and can cross-react with two envelope protein dimers on the virion surface)
		GFPDL	**594-** SYSLCTAAFTFTKIPAETLHGTVTVEVQYAGTDG PCKVPAQMAVDMQTLTPVGRLITANPVITESTEN SKMMLELDPPFGDSYIVIGVGEKKITHHWHRSG**-694**

Supplementary Figure 5: GFPDL based epitope mapping of neutralizing human MAb Z23. a) GFPDL-based epitope mapping of neutralizing ZIKV MAb Z23 to prME. b) The ELISA reactivity of selected GFPDL identified phage clones to MAb Z23 (highlighted in green, black and orange in a) was confirmed by phage ELISA and the ELISA positive clones are shown. c) Structure of GFPDL-identified epitope on PDB#5JHM for mature E colored blue on structure. d) The minimal sequence for MAb Z23 identified using GFPDL mapping (594-694, green in a) is shown in the table compared to the sequence previously identified 'known site' (Wang et. al., Sci. Trans. Med., 2016).

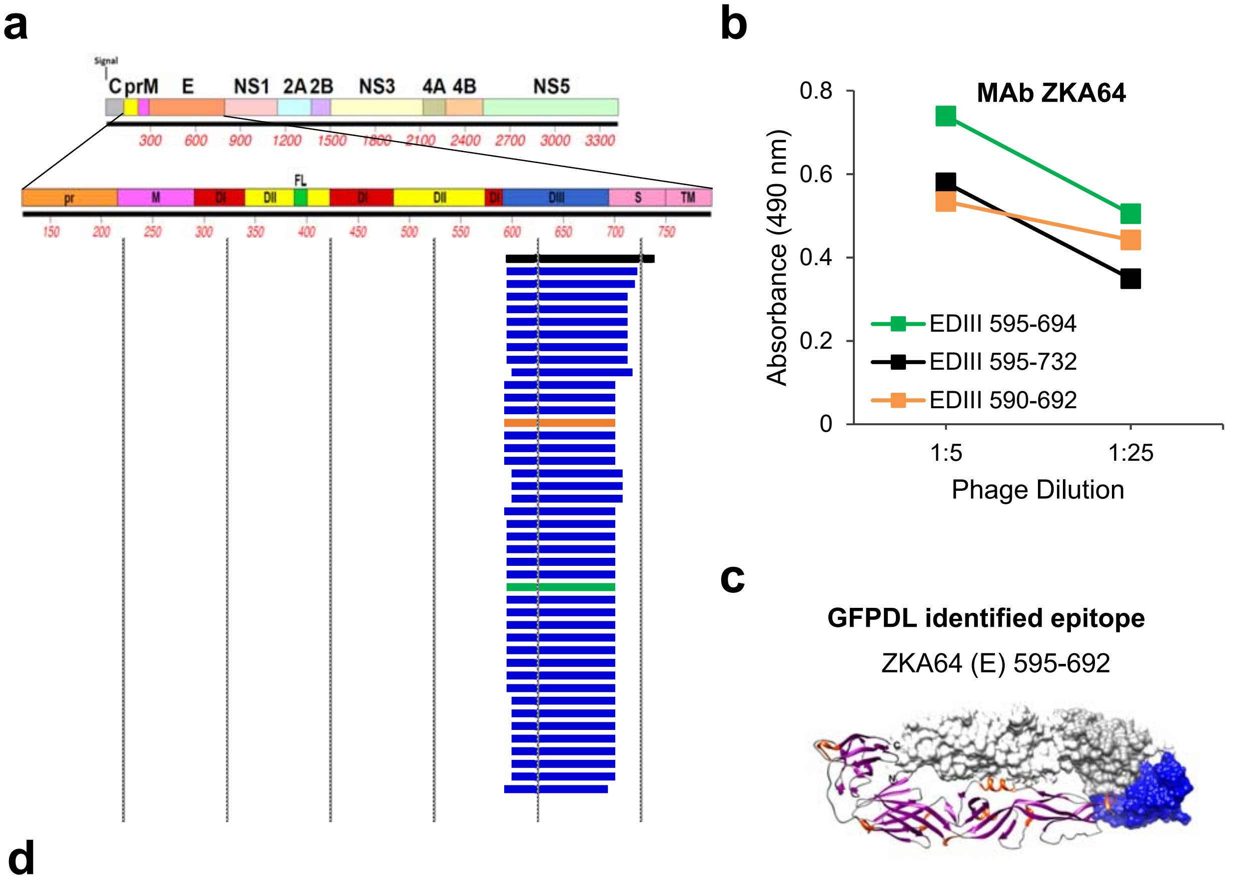

d

ZIKV MAb	Neutralizing Strains	Epitope	AA Sequence
ZKA64	Zika H/PF/2013, MR766	Known Site	ZIKV E DIII
		GFPDL	**595-** YSLCTAAFTFTKIPAETLHGTVTVEVQYAGTDG PCKVPAQMAVDMQTLTPVGRLITANPVITESTE NSKMMLELDPPFGDSYIVIGVGEKKITHHWHR- **692**

Supplementary Figure 6: GFPDL based epitope mapping of neutralizing human MAb ZKA64. a) GFPDL-based epitope mapping of neutralizing ZIKV MAb ZKA64 to prME. b) The ELISA reactivity of selected GFPDL identified phage clones to MAb ZKA64 (highlighted in green, black and orange in a) was confirmed by phage ELISA and the ELISA positive clones are shown. c) Structure of GFPDL-identified epitope on PDB#5JHM for mature E colored blue on structure. d) The minimal overlapping sequence for MAb ZKA64 identified using GFPDL mapping (595-692, green in a) is shown in the table compared to the sequence previously identified 'known site' (Stettler et. al., Science, 2016).

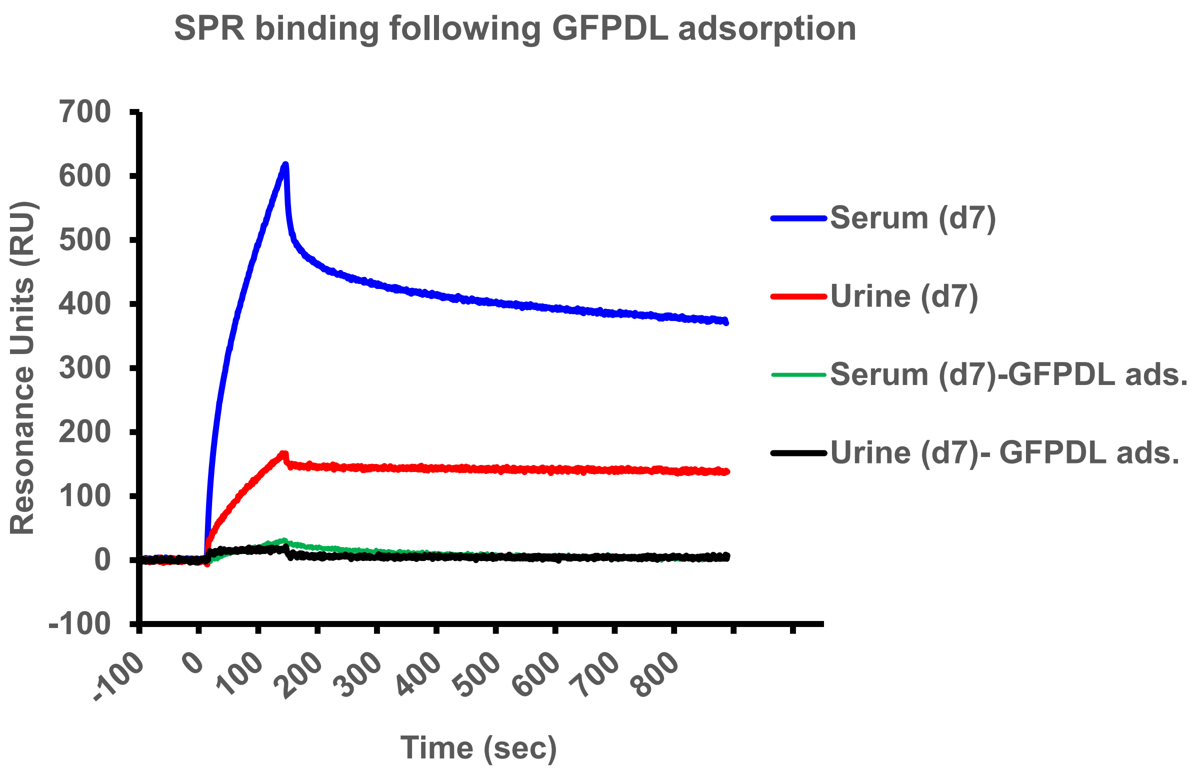

Supplementary Figure 7: Anti-E reactivity of post-infection sera or urine in SPR before and after ZIKV-GFPDL adsorption. Post infection sera or urine at day 7 from individuals was adsorbed on ZIKV-GFPDL coated petri dishes. Binding to recombinant ZIKV-E is shown before (Sera; blue and Urine; red lines) and after (Sera; green and Urine; black) GFPDL-adsorption in SPR.

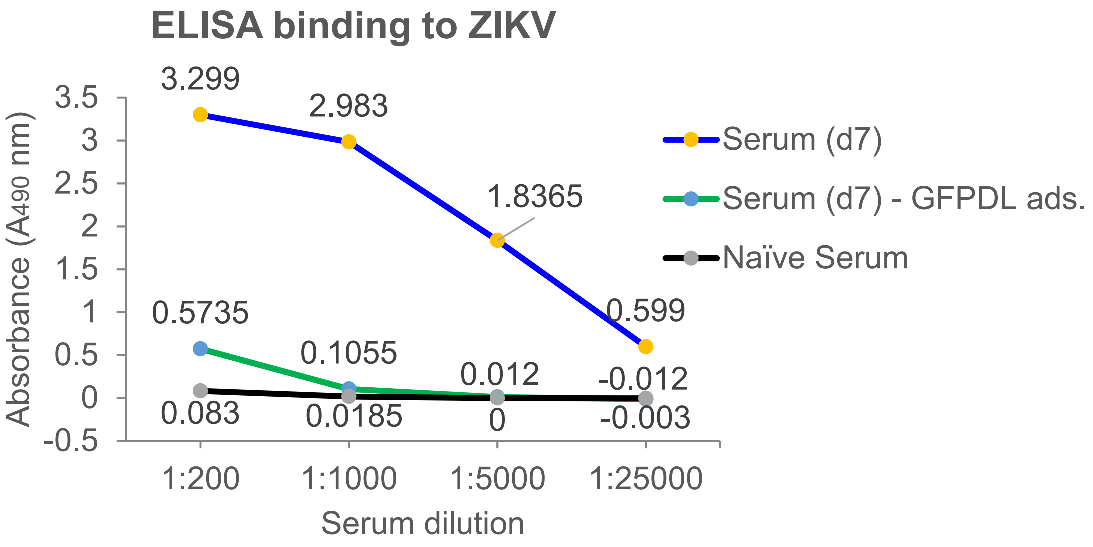

Supplementary Figure 8: Adsorption of anti-ZIKV antibodies in post-infection sera using ZIKV-GFPDL. Post ZIKV infection sera at day 7 (post-onset of symptoms) was adsorbed on ZIKV-GFPDL coated petri dishes. Antibody reactivity to Zika virus coated on polystyrene Immulon 2HB plates is shown before (blue line) and after (green line) GFPDL-adsorption in ELISA was revealed using HRP-conjugated goat anti-human IgA + IgG + IgM specific antibody. All data was normalized to 'virus only' background signal.

A

	IgM antibodies	IgG antibodies
Phage Titer	3.29×10^7	3.7×10^5

B **IgM antibodies**

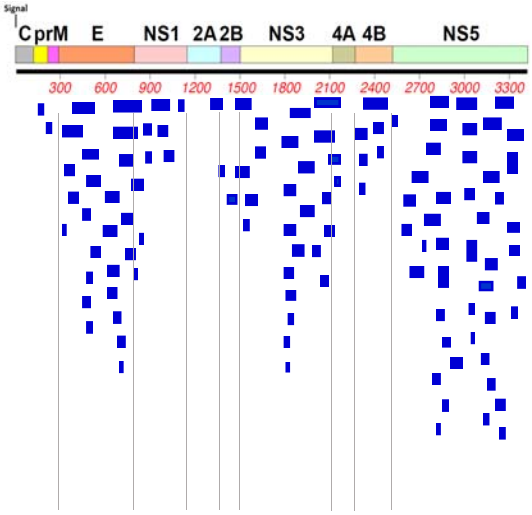

C **IgG antibodies**

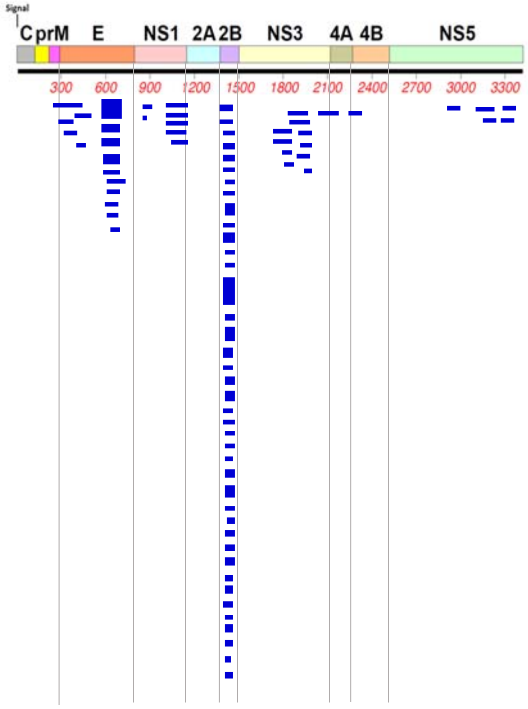

Supplementary Figure 9: Individual antibody repertoires elicited following acute ZIKV infection using IgG and IgM specific capture beads by ZIKV GFPDL. Alignment of bound phage clones to ZIKV genome. Schematic alignment of the peptide sequences recognized by IgM (A) and IgG (B) antibodies in ZIKV infected human sera (42-001-F at day 7 post-onset of illness), identified using ZIKV-GFPDL. The amino acid designation is based on the ZIKV polyprotein sequence encoded by the complete *ZIKV-ICD* genome (Supplementary Figure 1). Bars indicate identified peptides in the different structural (C, prM, E) and non-structural (NS) proteins on the ZIKV polyprotein sequence. Graphical distribution of representative clones with a frequency of $\geq$2, obtained after affinity selection, are shown. The horizontal position and the length of the bars indicate the peptide sequence displayed on the selected phage clone to its homologous sequence in the ZIKV sequence on alignment. The thickness of each bar represents the frequency of repetitively isolated phages.

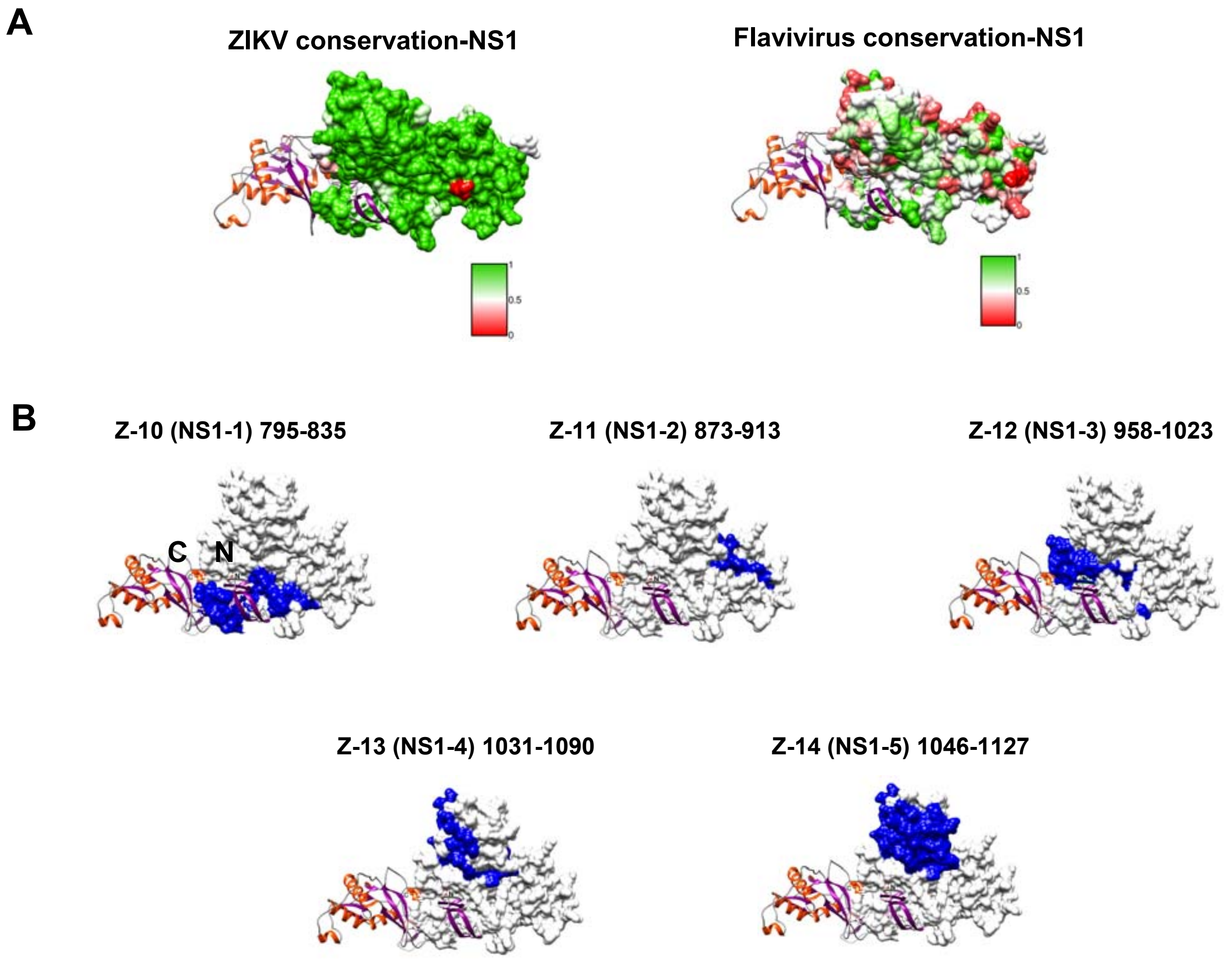

Supplementary Figure 10: Structural representation of antigenic sites identified in ZIKV NS1 protein using GFDPL. (A) Heat map on one monomer chain showing sequence conservation of various ZIKV (left panel; Paraiba, Uganda 1947, Nigeria 1968, Senegal 2001, Micronesia 2007 and Brazil 2016 strains) and flaviviruses (right panel; Dengue 1-4, West Nile, Yellow fever and ZIKV) conservation on ZIKV NS1 protein structure (PDB 5K6K). The heat map has been color coded from red (0) to green (1), where green denotes complete conservation. **b)** Antigenic sites have been depicted in blue on surface structures of ZIKV NS1 protein (PDB 5K6K). C- and N-terminals have been depicted on the first structure showing antigenic sites. Structure 5K6K encompasses residues 795-1146 on ZIKV_ICD whole genome sequence.

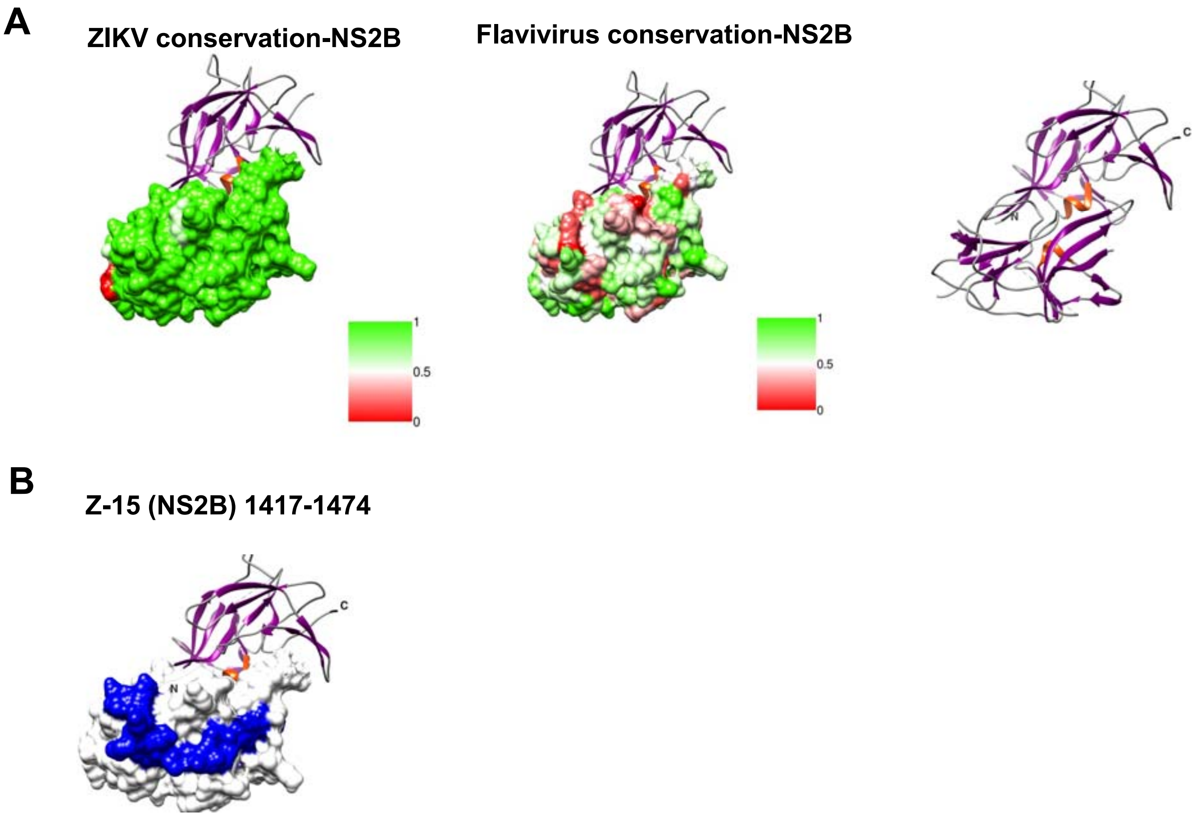

Supplementary Figure 11: Structural representation of antigenic sites identified in ZIKV NS2B protein using GFDPL. A) Heat map on one monomer chain showing various ZIKV (left panel; Paraiba/2015,MR766/Uganda/1947,Nigeria/IbH30656_SM21V1-V3/1968, ArD157995/Senegal/2001, Micronesia/2007 and Brazil/2015 strains) and flaviviruses (center panel; Dengue_1-4, West Nile, Yellow fever (and ZIKV) conservation on ZIKV NS2B-NS3 protein structure (PDB#5GXJ). The heat map has been color coded from red (0) to green (1), where green denotes complete conservation. C- and N-terminals have been depicted on the ribbon structure (right panel). B) Antigenic sites have been depicted in blue on surface structures of ZIKV NS2B protein (PDB#5GXJ). Structure PDB#5GXJ encompasses residues 1421-1673 on ZIKV ICD whole genome sequence.

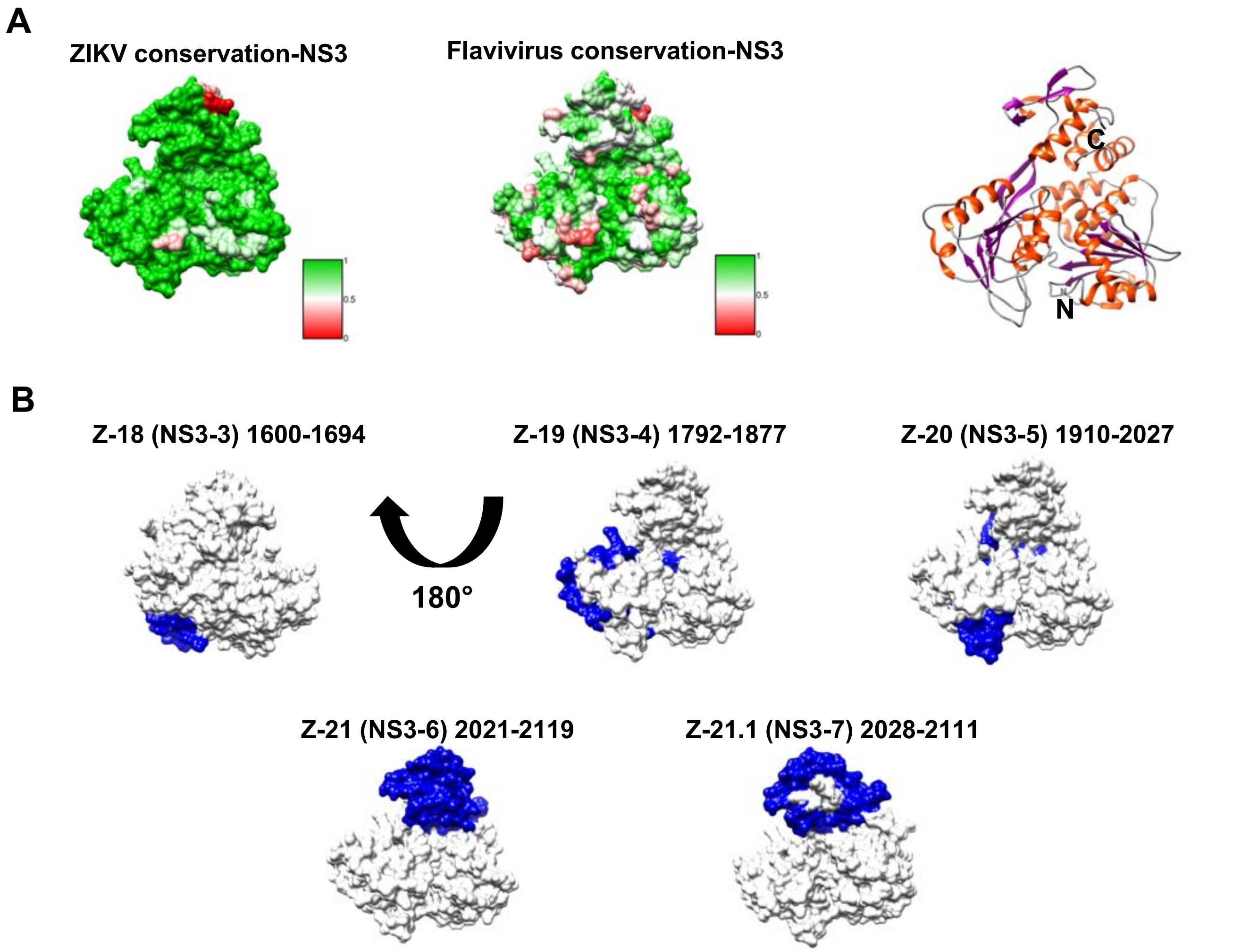

Supplementary Figure 12: Structural representation of antigenic sites identified in ZIKV NS3 protein using GFDPL. (A) Heat map showing various ZIKV (left panel; Paraiba, Uganda 1947, Nigeria 1968, Senegal 2001, Micronesia 2007 and Brazil 2016 strains) and flaviviruses (center panel; Dengue 1-4, West Nile virus, Yellow fever and ZIKV) conservation on ZIKV NS3 protein structure (PDB 5JRZ). The heat map has been color coded from red (0) to green (1), where green denotes complete conservation. C- and N- terminals have been depicted on the ribbon structure of 5JRZ (right panel). (B) Antigenic sites have been depicted in blue on surface structures of ZIKV NS3 protein (PDB 5JRZ). Structure 5JRZ encompasses residues 1677-2119 on ZIKV ICD whole genome sequence.

Supplementary Figure 13: Structural representation of antigenic sites identified in ZIKV NS5 protein using GFDPL. (A) Heat map showing various ZIKV (left panel; Paraiba, Uganda 1947, Nigeria 1968, Senegal 2001, Micronesia 2007 and Brazil 2016 strains) and flaviviruses (center panel; Dengue 1-4, West Nile virus, Yellow fever and ZIKV) conservation on ZIKV NS5 protein structure (PDB 5TFR). The heat map has been color coded from red (0) to green (1), where green denotes complete conservation. C- and N- terminals have been depicted on the ribbon structure of 5TFR (right panel). (B) Antigenic sites have been depicted in blue on surface structures of ZIKV NS5 protein (PDB 5TFR). Structure 5TFR encompasses residues 2525-3423 on ZIKV ICD whole genome sequence.

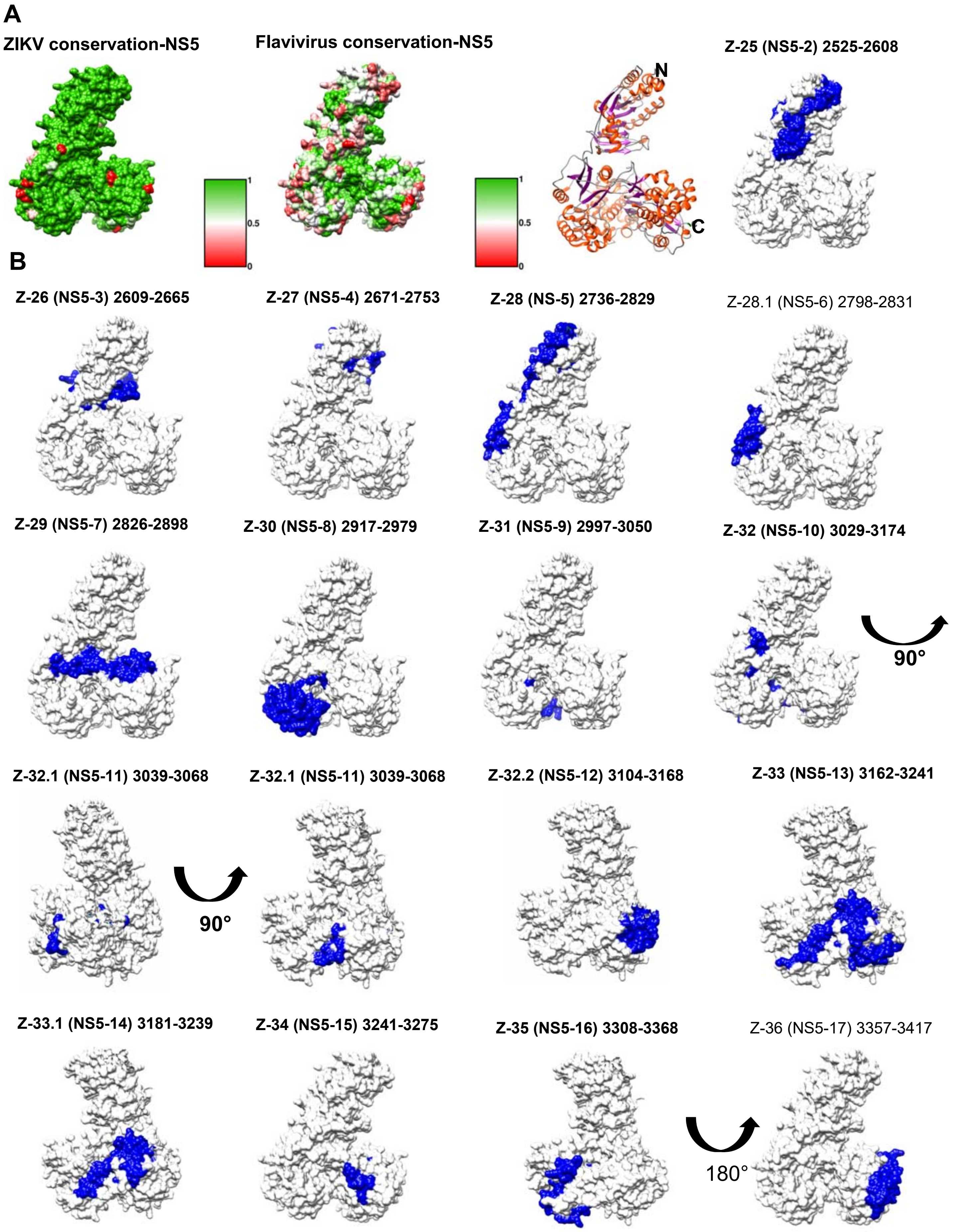
A
ZIKV conservation-NS5
Flavivirus conservation-NS5
1
0.5
0
N
C
Z-25 (NS5-2) 2525-2608
B
Z-26 (NS5-3) 2609-2665
Z-27 (NS5-4) 2671-2753
Z-28 (NS-5) 2736-2829
Z-28.1 (NS5-6) 2798-2831
Z-29 (NS5-7) 2826-2898
Z-30 (NS5-8) 2917-2979
Z-31 (NS5-9) 2997-3050
Z-32 (NS5-10) 3029-3174
90°
Z-32.1 (NS5-11) 3039-3068
Z-32.1 (NS5-11) 3039-3068
Z-32.2 (NS5-12) 3104-3168
Z-33 (NS5-13) 3162-3241
Z-33.1 (NS5-14) 3181-3239
Z-34 (NS5-15) 3241-3275
Z-35 (NS5-16) 3308-3368
180°
Z-36 (NS5-17) 3357-3417

Supplementary Figure 14: Percent similarity between different ZIKV strains and flaviviruses. (A) Similarity between various ZIKV strains plotted as a percentage of ZIKV Paraiba strain (considered at 100%) whose genome structure with antigenic sites has been depicted. (B) Similarity between various flaviviruses plotted as a percentage of ZIKV Paraiba strain (considered at 100%) whose genome structure with antigenic sites have been depicted. In both cases, ZIKV Paraiba strain was used as a query sequence and was used to generate a plot that shows the percent similarity of the reference sequences (other flaviviruses (panel A), ZIKV strains (panel B) to the query sequence. A sliding window of size 200 bp or 20 bp was used, which passes through the alignment in steps of 1 bp to generate the plot showing different flaviviruses and all ZIKV strains respectively.

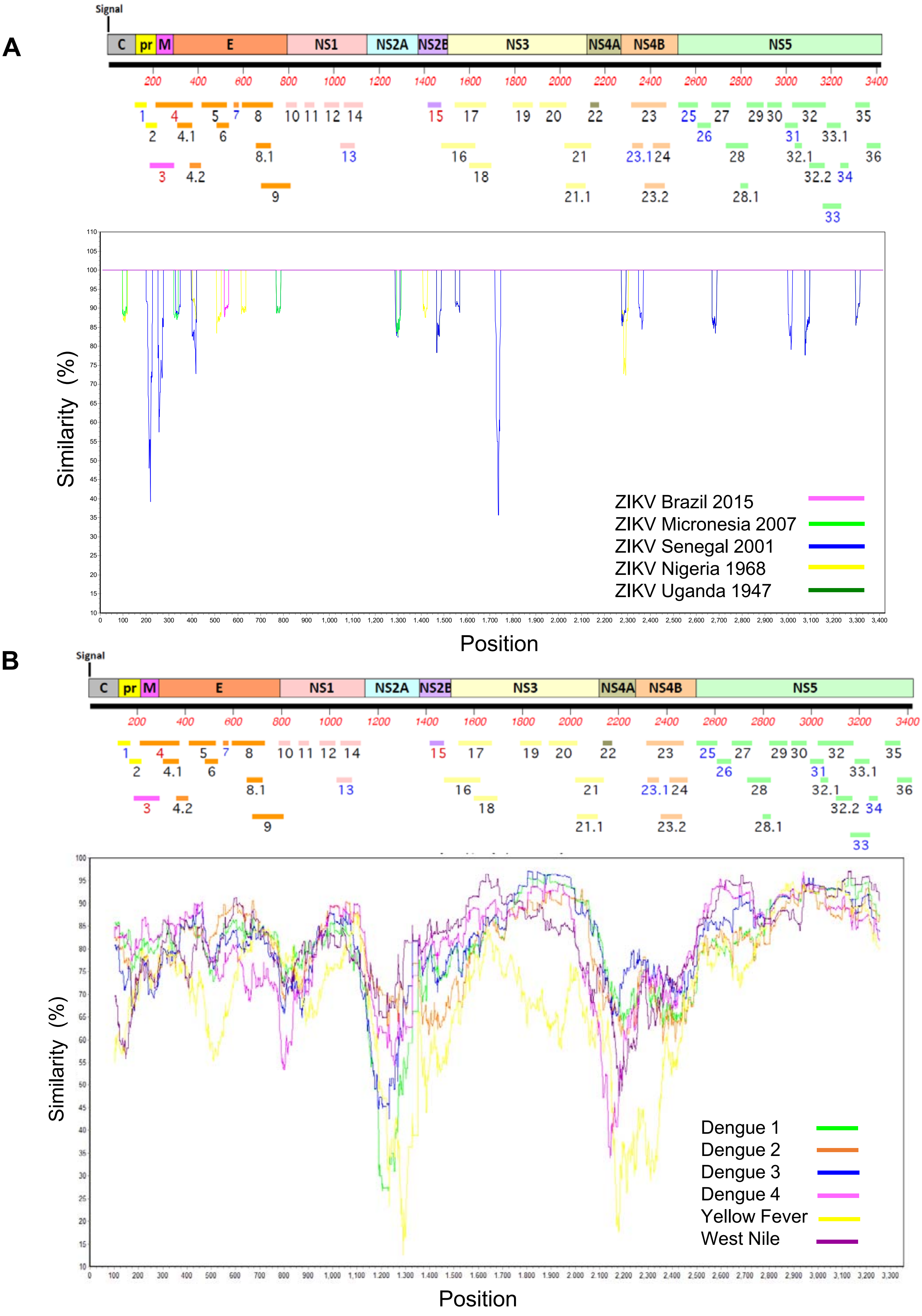
A
Signal
C
pr
M
E
NS1
NS2A
NS2B
NS3
NS4A
NS4B
NS5
Similarity (%)
Position
ZIKV Brazil 2015
ZIKV Micronesia 2007
ZIKV Senegal 2001
ZIKV Nigeria 1968
ZIKV Uganda 1947
B
Signal
C
pr
M
E
NS1
NS2A
NS2B
NS3
NS4A
NS4B
NS5
Similarity (%)
Position
Dengue 1
Dengue 2
Dengue 3
Dengue 4
Yellow Fever
West Nile

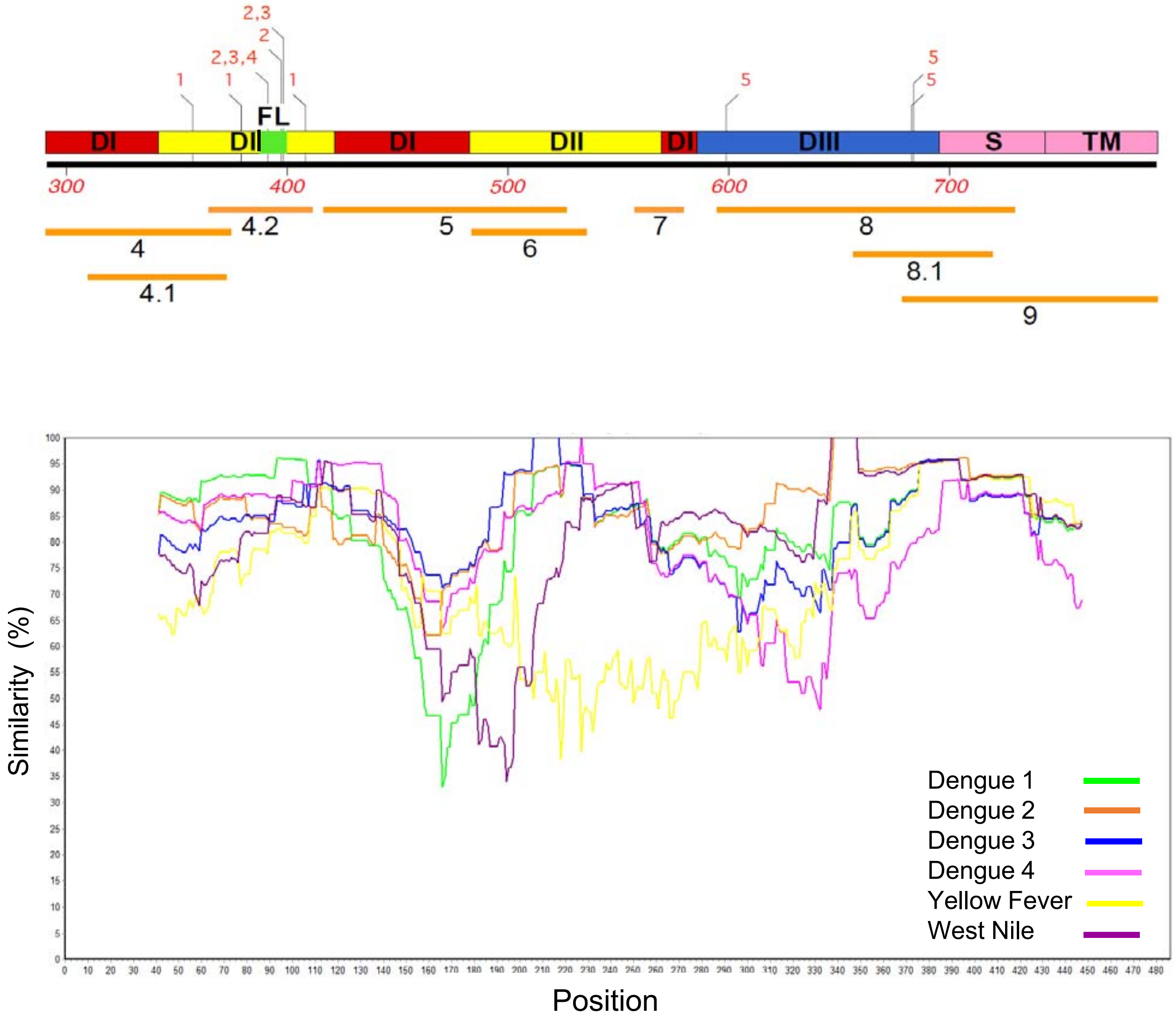

Supplementary Figure 15: Similarity plot of Envelope (E) protein sequence of different flaviviruses. For generating the plot, ZIKV_Paraiba strain was used as the query sequence to obtain the percent similarity of various domains of E protein of different flaviviruses to the ZIKV E. Highest conservation was seen near the fusion loop region while the lowest was seen around domain I (Antigenic sites 5,6,7) showing potential specificity to ZIKV E protein. A sliding window size of 80 bp and a step size of 1 bp was used to generate the plot.

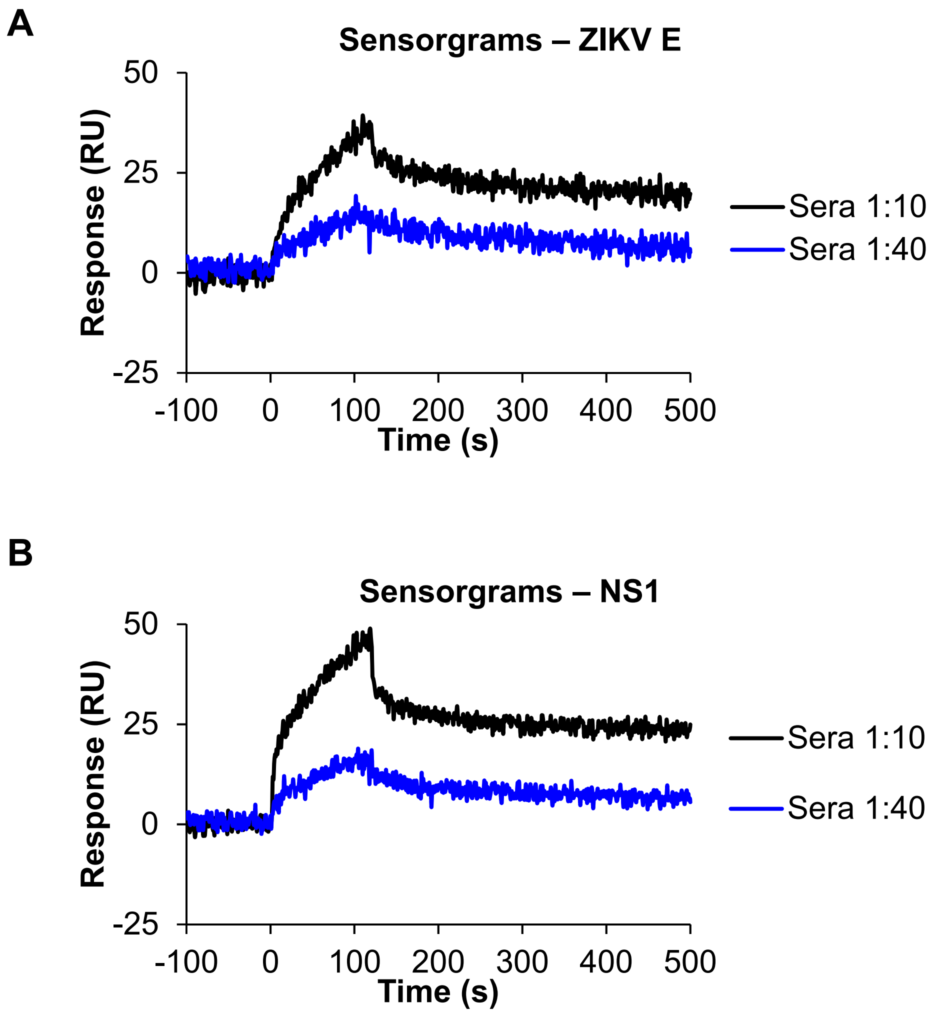

Supplementary Figure 16

Steady-state equilibrium analysis of different dilutions of post-infection sera to ZIKV-E and NS1 protein by SPR. Serial dilutions of post-infection sera were injected simultaneously onto either ZIKV-E (A) or NS1 (B) protein immobilized on a GLC sensor chip and on a surface free of protein (used as a blank). Binding was recorded using BioRad Proteon surface plasmon resonance biosensor instrument. Responses from the protein surface were corrected for the response from the mock surface and for responses from a separate, buffer only injection. Antibody off-rate constants, which describe the fraction of antigen-antibody complexes that decay per second, were determined directly from the serum sample interaction with ZIKV-E using SPR in the dissociation phase only for the sensorgrams with Max RU in the range of 10-100 RU (shown here for 10x and 40x fold dilution of sera) and calculated using the BioRad ProteOn manager software for the heterogeneous sample model

Supplementary Table 1: Demographic, epidemiological, serology and diagnostic information of samples used in the current study

Sample Characteristic						ELISA (Absorbance)^				PCR*					
ID	Visit (Day)	Gender	Age (Yr)	Prior DENV exposure	Days since Onset of Symptoms	ZIKV-IgG	ZIKV-IgM	DENV-IgG	DENV-IgM	ZIKV-Serum	ZIKV-Urine	DENV-Serum	DENV-Urine	Pan-Flavi-Serum	Pan-Flavi--Urine
41-001-F	0	F	41	NO	2	1.696	0.19	1.904	0.157	1	0	0	0	0	1
	3				5	0.019	0.536	0.019	0.127	0	1	0	0	0	1
	7				9	0.185	1.572	0.014	0.127	0	1	0	0	0	1
	28				30	0.755	0.995	0.044	0.139						
41-002-F	0	M	37	NO	2	1.222	0.022	0.797	0.116	0	0	0	0	0	0
	3				5	1.168	0.024	0.832	0.12	0	0	0	0	0	0
	7				9	1.137	0.029	0.774	0.117	1	0	0	0	1	0
	28				30	1.036	0.03	0.681	0.112						
41-003-F	0	M	51	NO	3	1.603	0.183	1.93	0.106	0	0	0	0	0	0
	3				6	1.519	0.14	1.788	0.09	1	0	0	0	1	0
	28				31	1.787	0.18	1.321	0.082						
41-006-F	0	M	49	NO	3	0.979	0.048	0.863	0.311	1	1	0	0	1	1
	3				6	2.569	0.292	2.185	0.373	1	1	0	0	1	1
	7				10	2.544	0.332	9.999	0.531	0	1	0	0	0	1
	28				31	2.494	0.245	9.999	0.457						
41-010-F	0	F	32	NO	3	0.171	0.079	0.602	0.082	0	1	0	0	0	1
	3				6	1.032	0.176	1.38	0.101	0	0	0	0	0	0
	7				10	1.943	0.635	9.999	0.246	0	0	0	0	0	0
	28				31	1.692	0.972	2.935	0.24						
41-017-F	0	F	34	NO	3	0.229	0.085	0.815	0.317	1	1	0	0	1	1
	3				6	1.162	0.271	1.861	0.363	0	1	0	0	0	1
	7				10	1.591	0.626	9.999	0.806	0	1	0	0	0	1
	28				31	2.282	0.231	9.999	0.635						
41-023-F	0	F	33	NO	0	0.83	0.152	1.358	0.211	1	1	0	0	0	0
	3				3	2.629	0.251	9.999	0.478	0	1	0	0	0	1
	7				7	2.684	0.348	9.999	0.685	0	1	0	0	0	0
	28				28	2.613	0.266	9.999	0.524						
41-028-F	0	M	38	NO	5	1.257	0.04	0.484	9.999	1	0	0	0	0	0
	3				8	2.445	0.168	9.999	9.999	0	1	0	0	0	0
	7				12	2.445	0.382	9.999	9.999	0	0	0	0	0	0
	28				33	2.024	0.122	2.851	9.999						
41-031-F	0	M	19	NO	2	1.858	0.037	0.374	0.04	0	1				
	3				5	2.576	0.062	0.593	0.059	0	1	0	0	0	0
	7				9	2.867	0.217	1.159	0.085	0	1	0	0	0	0
	28				30	2.678	0.045	1.022	0.075						

41-036-F	0	M	50	NO	3	1.32	0.566	1.583	0.135	0	1	0	0	0	1
	3				6	2.599	1.146	9.999	0.307	0	1	0	0	0	0
	7				10	2.486	0.734	9.999	0.295	0	1	0	0	0	0
	28				31	2.455	0.163	9.999	0.276						
42-001-F	0	F	35	NO	0	0.063	0.023	0.075	0.185	1	1	0	0	1	1
	3				3	1.417	0.142	1.396	0.578	0	1	0	0	0	1
	7				7	2.233	0.218	9.999	0.99	0	1	0	0	0	1
	28				28	1.218	0.137	2.187	0.401						
42-002-F	0	M	18	YES	0	0.893	0.028	1.591	0.178	0	0	0	0	0	0
	3				3	0.938	0.031	1.502	0.171	0	1	0	0	0	1
	7				7	0.915	0.025	1.437	0.179	0	0	0	0	0	0
	28				28	0.968	0.038	1.344	0.238						
42-003-F	0	M	37	NO	1	0.397	0.017	0.205	0.114	1	1	0	0	1	1
	3				4	1.8	0.025	0.45	0.194	0	1	0	0	0	1
	7				8	2.386	0.2	9.999	0.802	0	1	0	0	0	1
	28				29	2.172	0.135	9.999	1.049						
42-004-F	0	F	27	NO	5	1.663	0.057	1.556	0.079	0	1	0	0	0	0
	3				8	1.516	0.065	1.673	0.083	0	0	0	0	0	0
	7				12	1.452	0.039	1.486	0.072	0	0	0	0	0	0
	28				33	1.414	0.089	1.504	0.075						
42-007-F	0	F	24	NO	5	0.291	0.088	1.112	0.18	1	1	0	0	1	1
	3				8	0.956	0.111	1.505	0.222	0	1	0	0	0	1
	7				12	2.426	0.235	9.999	0.61	0		0		0	
	28				33	2.908	0.151	9.999	0.46						
42-008-F	0	M	35	NO	4	0.837	0.025	1.464	0.134	0	1			0	1
	3				7	2.32	0.041	9.999	0.395	0	1	0	0	0	0
	7				11	2.516	0.314	9.999	0.511	0	0	0	0	0	0
	28				32	9.999	0.154	9.999	0.39						
42-014-F	0	F	32	NO	5	0.091	0.337	1.762	0.661	1	1	0	0	0	0
	3				8	0.397	0.88	9.999	0.787	0	1	0	0	0	1
	7				12	0.954	0.553	9.999	0.795	0	1	0	0	0	0
	28				33	0.938	0.372	9.999	0.742						
42-018-F	0	F	33	NO	0	0.269	0.043	0.496	0.111	1	1	0	0	1	0
	3				3	1.425	0.086	0.812	0.128	1	1	0	0	0	1
	7				7	2.711	0.405	9.999	0.577	0	1	0	0	1	0
	28				28	2.289	0.125	9.999	0.346						
43-026-F	0	F	46	NO	3	1.768	0.109	0.669	0.235	1	1	0	0	0	1
	3				6	2.716	0.169	9.999	0.517	0	1	0	0	0	1
	7				10	2.79	0.156	9.999	0.696	0	1	0	0	0	1
	28				31	2.682	0.182	9.999	0.365						

^ Serologic assays were performed for ZIKA IgG and IgM antibodies by ELISA (Euroimmun), DENV IgG and IgM antibodies by ELISA (Panbio) using commercial diagnostic kits.

* PCR assays for Zika (recommended by WHO), Dengue and Pan-flavivirus were performed as described in Methods. PCR results are shown as: 1; postiive, and 0 ; negative.

Supplementary Table 2: Clinical Symptoms, collection dates and days since onset of symptoms for the acutely ZIKV infected patients

Sample Characteristic						Symptoms
ID	Visit (Day)	Gender	Age (Yr)	Date of Sample	Days since Onset of Symptoms	Number of symptoms[a]
41-001-F	0	F	41	6/21/2016	2	13
	3			6/23/2016	5	10
	7			6/28/2016	9	6
	28			7/22/2016	30	
41-002-F	0	M	37	6/22/2016	2	8
	3			6/24/2016	5	8
	7			6/29/2016	9	4
	28			7/20/2016	30	7
41-003-F	0	M	51	6/27/2016	3	11
	3			6/29/2016	6	14
	28			7/25/2016	31	8
41-006-F	0	M	49	7/5/2016	3	5
	3			7/8/2016	6	9
	7			7/13/2016	10	2
	28			8/1/2016	31	2
41-010-F	0	F	32	7/13/2016	3	13
	3			7/15/2016	6	5
	7			7/20/2016	10	4
	28			8/12/2016	31	2
41-017-F	0	F	34	8/8/2016	3	13
	3			8/10/2016	6	6
	7			8/15/2016	10	6
	28			9/5/2016	31	3
41-023-F	0	F	33	8/22/2016	0	16
	3			8/25/2016	3	10
	7			8/29/2016	7	2
	28			9/20/2016	28	6
41-028-F	0	M	38	9/6/2016	5	11
	3			9/9/2016	8	3
	7			9/14/2016	12	1
	28			10/5/2016	33	1
41-031-F	0	M	19	9/12/2016	2	12
	3			9/14/2016	5	13
	7			9/19/2016	9	5
	28			10/10/2016	30	11
41-036-F	0	M	50	10/10/2016	3	19
	3			10/13/2016	6	7
	7			10/17/2016	10	11
	28			11/9/2016	31	14
42-001-F	0	F	35	6/24/2016	0	17
	3			6/27/2016	3	15
	7			7/1/2016	7	12
	28			7/22/2016	28	7
42-002-F	0	M	18	6/29/2016	0	7
	3			7/1/2016	3	9
	7			7/6/2016	7	4
	28			7/29/2016	28	3

42-003-F	0<br>3<br>7<br>28	M	37	7/8/2016<br>7/11/2016<br>7/15/2016<br>8/5/2016	1<br>4<br>8<br>29	5<br>8<br>8<br>1
42-004-F	0<br>3<br>7<br>28	F	27	7/11/2016<br>7/15/2016<br>7/18/2016<br>8/8/2016	5<br>8<br>12<br>33	6<br>9<br>5<br>7
42-007-F	0<br>3<br>7<br>28	F	24	7/26/2016<br>7/29/2016<br>8/2/2016<br>8/23/2016	5<br>8<br>12<br>33	11<br>12<br>11<br>5
42-008-F	0<br>3<br>7<br>28	M	35	7/26/2016<br>7/29/2016<br>8/2/2016<br>8/23/2016	4<br>7<br>11<br>32	6<br>6<br>6
42-014-F	0<br>3<br>7<br>28	F	32	8/17/2016<br>8/19/2016<br>8/24/2016<br>9/14/2016	5<br>8<br>12<br>33	13<br>10<br>10<br>7
42-018-F	0<br>3<br>7<br>28	F	33	9/21/2016<br>9/23/2016<br>9/28/2016<br>10/20/2016	0<br>3<br>7<br>28	11<br>10<br>10<br>9
43-026-F	0<br>3<br>7<br>28	F	46	10/28/2016<br>10/31/2016<br>11/4/2016<br>11/25/2016	3<br>6<br>10<br>31	9<br>8<br>3<br>2

a- Symptom checklist included the following-Back pain, Bleeding, Confusion/disorientation, Conjunctivitis, Difficult standing upright / hunched, Difficulty walking, Fatigue, Headache, Itchiness, Joint ache (severe arthralgia), Malaise, Mouth ulcers, Muscle ache, Muscular weakness, Paresthesias, Periorbital pain, Photophobia, Rash, Sore throat

Supplementary Table 3: Frequency of antigenic sites for IgM and IgG antibodies in serum on day 0 and 7 and urine on day 7 post -ZIKV exposure.

			IgM			IgG		
**Antigenic Site**	**AA**	**Sequence**	**Serum D0**	**Serum D7**	**Urine D7**	**Serum D0**	**Serum D7**	**Urine D7**
Z-1 (pr) 122-170	122-170	AAEVTRRGSAYYMYLDRNDAGEAISFPTTLGMNKCYIQIMDLGHMCDAT	0%	1%	1%	0%	0%	0%
Z-2 (pr/M-1) 170-216	170-216	TMSYECPMLDEGVEPDDVDCWCNTTSTWVVYGTCHHKKGEARRSRRA	1%	1%	2%	1%	0%	16%
Z-3 (pr/M-2) 188-293	188-293	DCWCNTTSTWVVYGTCHHKKGEARRSRRAVTLPSHSTRKLQTRSQTWLESREYTKHLIRVENWIFRNP GFALAAAAIAWLLGSSTSQKVIYLVMILLIAPAYSIRC	0%	0%	0%	0%	1%	0%
Z-4 (M/E) 213-374	213-374	SRRAVTLPSHSTRKLQTRSQTWLESREYTKHLIRVENWIFRNPGFALAAAAIAWLLGSSTSQKVIYLVMIL LIAPAYSIRCIGVSNRDFVEGMSGGTWVDVVLEHGGCVTVMAQDKPTVDIELVTTTVSNMAEVRSYCY EASISDMASDSRCPTQGEAYLDK	0%	0%	0%	1%	1%	0%
Z-4.1 (E-1) 310-372	310-372	WVDVVLEHGGCVTVMAQDKPTVDIELVTTTVSNMAEVRSYCYEASISDMASDSRCPTQGEAYL	0%	1%	0%	4%	1%	0%
Z-4.2 (E-2) 365-411	365-411	PTQGEAYLDKQSDTQYVCKRTLVDRGWGNGCGLFGKGSLVTCAKFAC	2%	1%	0%	3%	2%	0%
Z-5 (E-3) 417-526	417-526	GKSIQPENLEYRIMLSVHGSQHSGMIVNDTGHETDENRAKVEITPNSPRAEATLGGFGSLGLDCEPRTG LDFSDLYYLTMNNKHWLVHKEWFHDIPLPWHAGADTGTPHW	1%	0%	0%	3%	0%	0%
Z-6 (E-4) 484-535	484-535	TGLDFSDLYYLTMNNKHWLVHKEWFHDIPLPWHAGADTGTPHWNNKEALVEF	3%	8%	3%	4%	1%	0%
Z-7 (E-5) 558-579	558-579	ALAGALEAEMDGAKGRLSSGHL	0%	0%	1%	0%	0%	0%
Z-8 (E-6) 595-729	595-729	YSLCTAAFTFTKIPAETLHGTVTVEVQYAGTDGPCKVPAQMAVDMQTLTPVGRLITANPVITESTENSK MMLELDPPFGDSYIVIGVGEKKITHHWHRSGSTIGKAFEATVRGAKRMAVLGDTAWDFGSVGGALN	1%	0%	0%	1%	16%	17%
Z-8.1 (E-7) 657-719	657-719	ESTENSKMMLELDPPFGDSYIVIGVGEKKITHHWHRSGSTIGKAFEATVRGAKRMAVLGDTAW	2%	1%	0%	7%	1%	0%
Z-9 (E-8) 679-794	679-806	IGVGEKKITHHWHRSGSTIGKAFEATVRGAKRMAVLGDTAWDFGSVGGALNSLGKGIHQIFGAAFKSL FGGMSWFSQILIGTLLMWLGLNTKNGSISLMCLALGGVLIFLSTAVSADVGCSVDFSKKE	0%	0%	2%	0%	1%	0%
Z-10 (NS1-1) 795-835	789-835	STAVSADVGCSVDFSKKETRCGTGVFVYNDVEAWRDRYKYHPDSPRR	1%	1%	1%	5%	0%	0%
Z-11 (NS1-2) 873-913	873-913	LEENGVQLTVVVGSVKNPMWRGPQRLPVPVNELPHGWKAWG	0%	1%	1%	0%	1%	0%
Z-12 (NS1-3) 958-1023	958-1023	HTSVWLKVREDYSLECDPAVIGTAVKGKEAVHSDLGYWIESEKNDTWRLKRAHLIEMKTCEWPKSH	2%	1%	0%	0%	1%	0%
Z-13 (NS1-4) 1031-1090	1031-1090	EESDLIIPKSLAGPLSHHNTREGYRTQMKGPWHSEELEIRFEECPGTKVHVEETCGTRGP	1%	1%	1%	0%	0%	0%
Z-14 (NS1-5) 1046-1127	1046-1127	SHHNTREGYRTQMKGPWHSEELEIRFEECPGTKVHVEETCGTRGPSLRSTTASGRVIEEWCCRECTMPP LSFRAKDGCWYGM	0%	1%	2%	5%	7%	0%
Z-15 (NS2B) 1417-1474	1417-1474	SGKSVDMYIERAGDITWEKDAEVTGNSPRLDVALDESGDFSLVEDDGPPMREIILKVV	0%	1%	0%	1%	49%	17%
Z-16 (NS3-1) 1503-1624	1477-1624	TICGMNPIAIPFAAGAWYVYVKTGKRSGALWDVPAPKEVKKGETTDGVYRVMTRRLLGSTQVGVGVM QEGVFHTMWHVTKGSALRSGEGRLDPYWGDVKQDLVSYCGPWKLDAAWDGHSEVQLLAVPPGERA RNIQTLPGIFKTKDGD	1%	0%	0%	1%	0%	0%
Z-17 (NS3-2) 1536-1672	1536-1672	TQVGVGVMQEGVFHTMWHVTKGSALRSGEGRLDPYWGDVKQDLVSYCGPWKLDAAWDGHSEVQ LLAVPPGERARNIQTLPGIFKTKDGDIGAVALDYPAGTSGSPILDKCGRVIGLYGNGVVIKNGSYVSAITQ GRR	3%	0%	1%	1%	0%	0%
Z-18 (NS3-3) 1600-1694	1600-1694	LAVPPGERARNIQTLPGIFKTKDGDIGAVALDYPAGTSGSPILDKCGRVIGLYGNGVVIKNGSYVSAITQG RREEETPVECFEPSMLKKKQLTVL	1%	0%	0%	1%	0%	0%
Z-19 (NS3-4) 1792-1877	1792-1877	TDPSSIAARGYISTRVEMGEAAAIFMTATPPGTRDAFPDSNSPIMDTEVEVPERAWSSGFDWVTDHSG KTVWFVPSVRNGNEIAAC	7%	14%	11%	7%	0%	17%
Z-20 (NS3-5) 1910-2027	1910-2027	TTDISEMGANFKADRVIDSRRCLKPVILDGERVILAGPMPVTHASAAQRRGRIGRNPNKPGDEYLYGGG CAETDEDHAHWLEARMLLDNIYLQDGLIASLYRPEADKVAAIEGEFKLR	2%	3%	1%	1%	0%	0%
Z-21 (NS3-6) 2021-2119	2021-2136	EGEFKLRTEQRKTFVELMKRGDLPVWLAYQVASAGITYTDRRWCFDGTTNNTIMEDSVPAEVWTRHG EKRVLKPRWMDARVCSDHAALKSFKEFAAGKRGAAFGVMEALGTLPGHM	1%	0%	0%	1%	1%	0%
Z-21.1 (NS3-7) 2028-2111	2028-2111	TEQRKTFVELMKRGDLPVWLAYQVASAGITYTDRRWCFDGTTNNTIMEDSVPAEVWTRHGEKRVLKP RWMDARVCSDHAALKSF	1%	2%	1%	1%	1%	0%
Z-22 (NS4A) 2135-2171	2135-2171	HMTERFQEAIDNLAVLMRAETGSRPYKAAAAQLPETL	1%	0%	0%	0%	0%	0%
Z-23 (NS4B-1) 2316-2468	2316-2468	TTFITPAVQHAVTTSYNNYSLMAMATQAGVLFGMGKGMPFYAWDFGVPLLMIGCYSQLTPLTLIVAIIL LVAHYMYLIPGLQAAAARAAQKRTAAGIMKNPVVDGIVVTDIDTMTIDPQVEKKMGQVLLIAVAVSSA ILSRTAWGWGEAGALI	2%	0%	0%	0%	1%	0%
Z-23.1 (NS4B-2) 2320-2365	2320-2365	TPAVQHAVTTSYNNYSLMAMATQAGVLFGMGKGMPFYAWDFGVPLL	0%	0%	2%	0%	0%	0%
Z-23.2 (NS4B-3) 2375-2462	2375-2462	PLTLIVAIILLVAHYMYLIPGLQAAAARAAQKRTAAGIMKNPVVDGIVVTDIDTMTIDPQVEKKMGQVLL IAVAVSSAILSRTAWGWG	1%	0%	1%	0%	1%	0%
Z-24 (NS5-1) 2413-2484	2413-2484	MKNPVVDGIVVTDIDTMTIDPQVEKKMGQVLLIAVAVSSAILSRTAWGWGEAGALITAATSTLWEGSP NKYW	1%	2%	3%	0%	0%	17%
Z-25 (NS5-2) 2525-2608	2525-2608	GETLGEKWKARLNQMSALEFYSYKKSGITEVCREEARRALKDGVATGGHAVSRGSAKLRWLVERGYLQ PYGKVIDLGCGRGGWS	1%	1%	0%	0%	0%	0%
Z-26 (NS5-3) 2609-2665	2609-2665	YYAATIRKVQEVKGYTKGGPGHEEPVLVQSYGWNIVRLKSGVDVFHMAAEPCDTLLC	1%	0%	3%	0%	0%	0%
Z-27 (NS5-4) 2671-2753	2671-2753	SSSPEVEEARTLRVLSMVGDWLEKRPGAFCIKVLCPYTSTMMETLERLQRRYGGGLVRVPLSRNSTHEM YWVSGAKSNTIKSV	2%	0%	1%	0%	1%	0%
Z-28 (NS-5) 2736-2829	2736-2829	THEMYWVSGAKSNTIKSVSTTSQLLLGRMDGPRRPVKYEEDVNLGSGTRAVVSCAEAPNMKIIGNRIER IRSEHAETWFFDENHPYRTWAYHGS	2%	1%	2%	1%	0%	0%
Z-28.1 (NS5-6) 2798-2831	2798-2831	IGNRIERIRSEHAETWFFDENHPYRTWAYHGSYE	5%	3%	2%	4%	1%	0%
Z-29 (NS5-7) 2826-2898	2826-2898	YHGSYEAPTQGSASSLINGVVRLLSKPWDVVTGVTGIAMTDTTPYGQQRVFKEKVDTRVPDPQEGTRQ VMSMV	2%	3%	6%	1%	0%	0%
Z-30 (NS5-8) 2917-2979	2917-2979	KEEFINKVRSNAALGAIFEEEKEWKTAVEAVNDPRFWALVDKEREHHLRGECQSCVYNMMGKR	3%	1%	2%	4%	1%	0%
Z-31 (NS5-9) 2997-3050	2997-3050	YMWLGARFLEFEALGFLNEDHWMGRENSGGGVEGLGLQRLGYVLEEMSRIPGGR	2%	0%	2%	0%	0%	0%
Z-32 (NS5-10) 3029-3174	3029-3174	EGLGLQRLGYVLEEMSRIPGGRMYADDTAGWDTRISRFDLENEALITNQMEKGHRALALAIIKYTYQNK VVKVLRPAEKGKTVMDIISRQDQRGSGQVVTYALNTFTNLVVQLIRNMEAEEVLEMQDLWLLRRSEKV TNWLQSNGW	1%	0%	1%	1%	0%	0%
Z-32.1 (NS5-11) 3039-3068	3039-3068	VLEEMSRIPGGRMYADDTAGWDTRISRFDL	2%	1%	1%	4%	0%	0%
Z-32.2 (NS5-12) 3104-3168	3104-3168	PAEKGKTVMDIISRQDQRGSGQVVTYALNTFTNLVVQLIRNMEAEEVLEMQDLWLLRRSEKVTNW	1%	9%	2%	0%	0%	16%
Z-33 (NS5-13) 3162-3241	3162-3241	SEKVTNWLQSNGWDRLKRMAVSGDDCVVKPIDDRFAHALRFLNDMGKVRKDTQEWKPSTGWDNW EEVPFCSHHFNKLHLK	2%	3%	0%	0%	0%	0%
Z-33.1 (NS5-14) 3181-3239	3181-3239	AVSGDDCVVKPIDDRFAHALRFLNDMGKVRKDTQEWKPSTGWDNWEEVPFCSHHFNKLH	2%	2%	2%	4%	1%	0%
Z-34 (NS5-15) 3241-3275	3241-3275	KDGRSIVVPCRHQDELIGRARVSPGAGWSIRETAC	0%	0%	2%	0%	0%	0%
Z-35 (NS5-16) 3308-3368	3308-3368	DWVPTGRTTWSIHGKGEWMTTEDMLVVWNRVWIEENDHMEDKTPVTKWTDIPYLGKREDLW	2%	6%	6%	3%	0%	0%
Z-36 (NS5-17) 3357-3417	3357-3417	DIPYLGKREDLWCGSLIGHRPRTTWAENIKNTVNMVRRIIGDEEKYMDYLSTQVRYLGEEG	0%	1%	2%	3%	1%	0%
		**Total***	**63%**	**71%**	**68%**	**78%**	**91%**	**100%**

* Total percentage of clones are comprised of clones represented in these antigenic sites for the analyzed sample.
The remaining clones are not represented by any unique antigenic site as clonal frequency is less than 2 for all analyzed samples

Supplementary Table 4: Sequence conservation of Antigenic regions/sites among different Flavivirus strains

Antigenic Site	AA	Sequence	Similarity of ZIKV antigenic Sites to other flaviviruses (%)					
			Dengue 1	Dengue 2	Dengue 3	Dengue 4	Yellow Fever	West Nile
Z-1 (pr) 122-170	122-170	AAEVTRRGSAYYMYLDRNDAGEAISFPTTLGMNKCYIQIMDLGHMCDAT	35%	35%	39%	37%	14%	29%
Z-2 (pr/M-1) 170-216	170-216	TMSYECPMLDEGVEPDDVDCWCNTTSTWVVYGTCHHKKGEARRSRRA	62%	57%	60%	57%	50%	51%
Z-3 (pr/M-2) 188-293	188-293	DCWCNTTSTWVVYGTCHHKKGEARRSRRAVTLPSHSTRKLQTRSQTWLESREYTKHLIRVENWIFRNPGFALAAAAIAWLLGSSTSQKVIYLVMILLIAPAYSIRC	43%	43%	43%	51%	42%	47%
Z-4 (M/E) 213-374	213-374	SRRAVTLPSHSTRKLQTRSQTWLESREYTKHLIRVENWIFRNPGFALAAAAIAWLLGSSTSQKVIYLVMILLIAPAYSIRCIGVSNRDFVEGMSGGTWVDVVLEHGGCVTVMAQDKPTVDIELVTTTVSNMAEVRSYCYEASISDMASDSRCPTQGEAYLDK	51%	47%	49%	55%	46%	51%
Z-4.1 (E-1) 310-372	310-372	WVDVVLEHGGCVTVMAQDKPTVDIELVTTTVSNMAEVRSYCYEASISDMASDSRCPTQGEAYL	68%	55%	62%	65%	48%	52%
Z-4.2 (E-2) 365-411	365-411	PTQGEAYLDKQSDTQYVCKRTLVDRGWGNGCGLFGKGSLVTCAKFAC	77%	66%	79%	72%	64%	70%
Z-5 (E-3) 417-526	417-526	GKSIQPENLEYRIMLSVHGSQHSGMIVNDTGHETDENRAKVEITPNSPRAEATLGGFGSLGLDCEPRTGLDFSDLYYLTMNNKHWLVHKEWFHDIPLPWHAGADTGTPHW	47%	47%	48%	46%	22%	35%
Z-6 (E-4) 484-535	484-535	TGLDFSDLYYLTMNNKHWLVHKEWFHDIPLPWHAGADTGTPHWNNKEALVEF	56%	58%	62%	56%	35%	42%
Z-7 (E-5) 558-579	558-579	ALAGALEAEMDGAKGRLSSGHL	41%	45%	41%	41%	32%	50%
Z-8 (E-6) 595-729	595-729	YSLCTAAFTFTKIPAETLHGTVTVEVQYAGTDGPCKVPAQMAVDMQTLTPVGRLITANPVITESTENSKMMLELDPPFGDSYIVIGVGEKKITHHWHRSGSTIGKAFEATVRGAKRMAVLGDTAWDFGSVGGALN	55%	52%	53%	54%	42%	62%
Z-8.1 (E-7) 657-719	657-719	ESTENSKMMLELDPPFGDSYIVIGVGEKKITHHWHRSGSTIGKAFEATVRGAKRMAVLGDTAW	56%	56%	52%	65%	48%	68%
Z-9 (E-8) 679-794	679-806	IGVGEKKITHHWHRSGSTIGKAFEATVRGAKRMAVLGDTAWDFGSVGGALNSLGKGIHQIFGAAFKSLFGGMSWFSQILIGTLLMWLGLNTKNGSISLMCLALGGVLIFLSTAVSADVGCSVDFSKKE	55%	48%	57%	56%	48%	62%
Z-10 (NS1-1) 795-835	789-835	STAVSADVGCSVDFSKKETRCGTGVFVYNDVEAWRDRYKYHPDSPRR	43%	40%	45%	45%	40%	55%
Z-11 (NS1-2) 873-913	873-913	LEENGVQLTVVVGSVKNPMWRGPQRLPVPVNELPHGWKAWG	37%	41%	41%	46%	41%	46%
Z-12 (NS1-3) 958-1023	958-1023	HTSVWLKVREDYSLECDPAVIGTAVKGKEAVHSDLGYWIESEKNDTWRLKRAHLIEMKTCEWPKSH	58%	50%	58%	53%	34%	58%
Z-13 (NS1-4) 1031-1090	1031-1090	EESDLIIPKSLAGPLSHHNTREGYRTQMKGPWHSEELEIRFEECPGTKVHVEETCGTRGP	57%	60%	63%	60%	45%	57%
Z-14 (NS1-5) 1046-1127	1046-1127	SHHNTREGYRTQMKGPWHSEELEIRFEECPGTKVHVEETCGTRGPSLRSTTASGRVIEEWCCRECTMPPLSFRAKDGCWYGM	62%	65%	62%	65%	57%	55%
Z-15 (NS2B) 1417-1474	1417-1474	SGKSVDMYIERAGDITWEKDAEVTGNSPRLDVALDESGDFSLVEDDGPPMREIILKVV	29%	33%	34%	34%	31%	55%
Z-16 (NS3-1) 1503-1624	1477-1624	TICGMNPIAIPFAAGAWYVYVKTGKRSGALWDVPAPKEVKKGETTDGVYRVMTRRLLGSTQVGVGVMQEGVFHTMWHVTKGSALRSGEGRLDPYWGDVKQDLVSYCGPWKLDAAWDGHSEVQLLAVPPGERARNIQTLPGIFKTKDGD	51%	49%	49%	51%	44%	60%
Z-17 (NS3-2) 1536-1672	1536-1672	TQVGVGVMQEGVFHTMWHVTKGSALRSGEGRLDPYWGDVKQDLVSYCGPWKLDAAWDGHSEVQLLAVPPGERARNIQTLPGIFKTKDGDIGAVALDYPAGTSGSPILDKCGRVIGLYGNGVVIKNGSYVSAITQGRR	56%	58%	59%	60%	57%	58%
Z-18 (NS3-3) 1600-1694	1600-1694	LAVPPGERARNIQTLPGIFKTKDGDIGAVALDYPAGTSGSPILDKCGRVIGLYGNGVVIKNGSYVSAITQGRREEETPVECFEPSMLKKKQLTVL	49%	53%	54%	55%	55%	65%
Z-19 (NS3-4) 1792-1877	1792-1877	TDPSSIAARGYISTRVEMGEAAAIFMTATPPGTRDAFPDSNSPIMDTEVEVPERAWSSGFDWVTDHSGKTVWFVPSVRNGNEIAAC	72%	77%	74%	77%	48%	71%
Z-20 (NS3-5) 1910-2027	1910-2027	TTDISEMGANFKADRVIDSRRCLKPVILDGERVILAGPMPVTHASAAQRRGRIGRNPNKPGDEYLYGGGCAETDEDHAHWLEARMLLDNIYLQDGLIASLYRPEADKVAAIEGEFKLR	73%	71%	71%	70%	49%	63%
Z-21 (NS3-6) 2021-2119	2021-2136	EGEFKLRTEQRKTFVELMKRGDLPVWLAYQVASAGITYTDRRWCFDGTTNNTIMEDSVPAEVWTRHGEKRVLKPRWMDARVCSDHAALKSFKEFAAGKRGAAFGVMEALGTLPGHM	57%	60%	58%	59%	47%	59%
Z-21.1 (NS3-7) 2028-2111	2028-2111	TEQRKTFVELMKRGDLPVWLAYQVASAGITYTDRRWCFDGTTNNTIMEDSVPAEVWTRHGEKRVLKPRWMDARVCSDHAALKSF	43%	54%	43%	38%	32%	35%
Z-22 (NS4A) 2135-2171	2135-2171	HMTERFQEAIDNLAVLMRAETGSRPYKAAAAQLPETL	52%	55%	52%	50%	31%	42%
Z-23 (NS4B-1) 2316-2468	2316-2468	TTFITPAVQHAVTTSYNNYSLMAMATQAGVLFGMGKGMPFYAWDFGVPLLMIGCYSQLTPLTLIVAIILLVAHYMYLIPGLQAAAARAAQKRTAAGIMKNPVVDGIVVTDIDTMTIDPQVEKKMGQVLLIAVAVSSAILSRTAWGWGEAGALI	47%	49%	47%	44%	34%	42%
Z-23.1 (NS4B-2) 2320-2365	2320-2365	TPAVQHAVTTSYNNYSLMAMATQAGVLFGMGKGMPFYAWDFGVPLL	46%	52%	50%	50%	37%	37%
Z-23.2 (NS4B-3) 2375-2462	2375-2462	PLTLIVAIILLVAHYMYLIPGLQAAAARAAQKRTAAGIMKNPVVDGIVVTDIDTMTIDPQVEKKMGQVLLIAVAVSSAILSRTAWGWG	55%	55%	52%	50%	30%	44%
Z-24 (NS5-1) 2413-2484	2413-2484	MKNPVVDGIVVTDIDTMTIDPQVEKKMGQVLLIAVAVSSAILSRTAWGWGEAGALITAATSTLWEGSPNKYW	47%	49%	47%	44%	34%	42%
Z-25 (NS5-2) 2525-2608	2525-2608	GETLGEKWKARLNQMSALEFYSYKKSGITEVCREEARRALKDGVATGGHAVSRGSAKLRWLVERGYLQPYGKVIDLGCGRGGWS	69%	67%	70%	67%	57%	64%
Z-26 (NS5-3) 2609-2665	2609-2665	YYAATIRKVQEVKGYTKGGPGHEEPVLVQSYGWNIVRLKSGVDVFHMAAEPCDTLLC	67%	65%	69%	67%	51%	77%
Z-27 (NS5-4) 2671-2753	2671-2753	SSSPEVEEARTLRVLSMVGDWLEKRPGAFCIKVLCPYTSTMMETLERLQRRYGGGLVRVPLSRNSTHEMYWVSGAKSNTIKSV	58%	60%	63%	66%	57%	69%
Z-28 (NS-5) 2736-2829	2736-2829	THEMYWVSGAKSNTIKSVSTTSQLLLGRMDGPRRPVKYEEDVNLGSGTRAVVSCAEAPNMKIIGNRIERIRSEHAETWFFDENHPYRTWAYHGS	53%	52%	52%	57%	49%	57%
Z-28.1 (NS5-6) 2798-2831	2798-2831	IGNRIERIRSEHAETWFFDENHPYRTWAYHGSYE	68%	65%	65%	65%	56%	68%
Z-29 (NS5-7) 2826-2898	2826-2898	YHGSYEAPTQGSASSLINGVVRLLSKPWDVVTGVTGIAMTDTTPYGQQRVFKEKVDTRVPDPQEGTRQVMSMV	70%	70%	71%	73%	62%	70%
Z-30 (NS5-8) 2917-2979	2917-2979	KEEFINKVRSNAALGAIFEEEKEWKTAVEAVNDPRFWALVDKEREHHLRGECQSCVYNMMGKR	63%	71%	63%	71%	68%	71%
Z-31 (NS5-9) 2997-3050	2997-3050	YMWLGARFLEFEALGFLNEDHWMGRENSGGGVEGLGLQRLGYVLEEMSRIPGGR	74%	72%	74%	76%	74%	80%
Z-32 (NS5-10) 3029-3174	3029-3174	EGLGLQRLGYVLEEMSRIPGGRMYADDTAGWDTRISRFDLENEALITNQMEKGHRALALAIIKYTYQNKVVKVLRPAEKGKTVMDIISRQDQRGSGQVVTYALNTFTNLVVQLIRNMEAEEVLEMQDLWLLRRSEKVTNWLQSNGW	60%	60%	62%	62%	57%	62%
Z-32.1 (NS5-11) 3039-3068	3039-3068	VLEEMSRIPGGRMYADDTAGWDTRISRFDL	70%	63%	70%	63%	57%	63%
Z-32.2 (NS5-12) 3104-3168	3104-3168	PAEKGKTVMDIISRQDQRGSGQVVTYALNTFTNLVVQLIRNMEAEEVLEMQDLWLLRRSEKVTNW	52%	58%	54%	55%	58%	64%
Z-33 (NS5-13) 3162-3241	3162-3241	SEKVTNWLQSNGWDRLKRMAVSGDDCVVKPIDDRFAHALRFLNDMGKVRKDTQEWKPSTGWDNWEEVPFCSHHFNKLHLK	68%	65%	68%	65%	66%	67%
Z-33.1 (NS5-14) 3181-3239	3181-3239	AVSGDDCVVKPIDDRFAHALRFLNDMGKVRKDTQEWKPSTGWDNWEEVPFCSHHFNKLH	73%	71%	73%	71%	71%	75%
Z-34 (NS5-15) 3241-3275	3241-3275	KDGRSIVVPCRHQDELIGRARVSPGAGWSIRETAC	86%	83%	83%	86%	83%	80%
Z-35 (NS5-16) 3308-3368	3308-3368	DWVPTGRTTWSIHGKGEWMTTEDMLVVWNRVWIEENDHMEDKTPVTKWTDIPYLGKREDLW	77%	79%	77%	75%	75%	81%
Z-36 (NS5-17) 3357-3417	3357-3417	DIPYLGKREDLWCGSLIGHRPRTTWAENIKNTVNMVRRIIGDEEKYMDYLSTQVRYLGEEG	54%	57%	52%	57%	46%	57%
