# Supplementary material for: Differential human antibody repertoires following Zika infection and the implications for serodiagnostics and disease outcome
Source: Nat Commun. 2019 Apr 26;10:1943. doi: 10.1038/s41467-019-09914-3 (PMC6486612; doi:10.1038/s41467-019-09914-3)
Supplement: Supplementary file 3 — Reporting Summary [file 41467_2019_9914_MOESM3_ESM.pdf]

nature research

Corresponding author(s): NCOMMS-18-26824-A

Reporting Summary

Nature Research wishes to improve the reproducibility of the work that we publish. This form provides structure for consistency and transparency in reporting. For further information on Nature Research policies, see Authors & Referees and the Editorial Policy Checklist.

Statistical parameters

When statistical analyses are reported, confirm that the following items are present in the relevant location (e.g. figure legend, table legend, main text, or Methods section).

n/a	Confirmed	
☐	☒	The exact sample size (*n*) for each experimental group/condition, given as a discrete number and unit of measurement
☐	☒	An indication of whether measurements were taken from distinct samples or whether the same sample was measured repeatedly
☐	☒	The statistical test(s) used AND whether they are one- or two-sided<br>*Only common tests should be described solely by name; describe more complex techniques in the Methods section.*
☒	☐	A description of all covariates tested
☐	☒	A description of any assumptions or corrections, such as tests of normality and adjustment for multiple comparisons
☐	☒	A full description of the statistics including central tendency (e.g. means) or other basic estimates (e.g. regression coefficient) AND variation (e.g. standard deviation) or associated estimates of uncertainty (e.g. confidence intervals)
☒	☐	For null hypothesis testing, the test statistic (e.g. *F*, *t*, *r*) with confidence intervals, effect sizes, degrees of freedom and *P* value noted<br>*Give P values as exact values whenever suitable.*
☒	☐	For Bayesian analysis, information on the choice of priors and Markov chain Monte Carlo settings
☒	☐	For hierarchical and complex designs, identification of the appropriate level for tests and full reporting of outcomes
☐	☒	Estimates of effect sizes (e.g. Cohen's *d*, Pearson's *r*), indicating how they were calculated
☐	☒	Clearly defined error bars<br>*State explicitly what error bars represent (e.g. SD, SE, CI)*

Our web collection on statistics for biologists may be useful.

Software and code

Policy information about availability of computer code

Data collection	BioRad ProteON Manager software (Version 3.1.0) to collect antibody binding data from SPR machine (www.Biorad.com)
Data analysis	BioRad ProteON Manager software (Version 3.1.0) for antibody binding analysis from SPR machine (www.Biorad.com)

For manuscripts utilizing custom algorithms or software that are central to the research but not yet described in published literature, software must be made available to editors/reviewers upon request. We strongly encourage code deposition in a community repository (e.g. GitHub). See the Nature Research guidelines for submitting code & software for further information.

Data

Policy information about availability of data

All manuscripts must include a data availability statement. This statement should provide the following information, where applicable:

- Accession codes, unique identifiers, or web links for publicly available datasets
- A list of figures that have associated raw data
- A description of any restrictions on data availability

The datasets generated during and/or analyzed during the current study are available from the corresponding author on reasonable request. The source data underlying Figs 1, 2, 5, 6 and 7 are provided in Supplementary tables 1-4 and as a Source Data file.

Field-specific reporting

Please select the best fit for your research. If you are not sure, read the appropriate sections before making your selection.

- [x] Life sciences
- [ ] Behavioural & social sciences

For a reference copy of the document with all sections, see nature.com/authors/policies/ReportingSummary-flat.pdf

Life sciences

Study design

All studies must disclose on these points even when the disclosure is negative.

Sample size	All samples from the Zika infected individuals were analyzed in this study
Data exclusions	No data was excluded
Replication	GFPDL and SPR analysis were performed twice by independent researchers in the lab
Randomization	All samples from the Zika infected individuals were analyzed in this study. There are no differential groups or arms in this study.
Blinding	Experiments were performed by different investigators, who were blinded to sample identity.

Materials & experimental systems

Policy information about availability of materials

n/a	Involved in the study	
☒	☐	Unique materials
☒	☐	Antibodies
☒	☐	Eukaryotic cell lines
☒	☐	Research animals
☐	☒	Human research participants

Human research participants

Policy information about studies involving human research participants

Population characteristics	Individuals of all ages and any gender that met the 12 February 2016 WHO/PAHO case definition (World Health Organization. Zika virus disease: Interim case definitions http://apps.who.int/iris/handle/10665/204381) of suspected acute Zika (fever and/or rash, and one or more other symptoms including arthralgia, myalgia, non-purulent conjunctivitis or conjunctival hyperemia, headache, and malaise) were eligible for the study.

Method-specific reporting

n/a	Involved in the study	
☒	☐	ChIP-seq
☒	☐	Flow cytometry
☒	☐	Magnetic resonance imaging
